# The scar that takes time to heal: A systematic review of COVID-19-related stigma targets, antecedents, and outcomes

**DOI:** 10.3389/fpsyg.2022.1026712

**Published:** 2022-12-01

**Authors:** Xiang Zhou, Chen Chen, Yuewei Yao, Jingtian Xia, Limei Cao, Xin Qin

**Affiliations:** Department of Business Administration, Sun Yat-sen University, Guangzhou, China

**Keywords:** COVID-19, stigma, Chinese/Asian people, patients and survivors, healthcare workers

## Abstract

COVID-19, as a crucial public health crisis, has affected our lives in nearly every aspect. Besides its major health threats, COVID-19 brings severe secondary impacts, one of which is the rise of social stigma. Although numerous studies have examined the antecedents and outcomes of COVID-19-related stigma, we still lack a systematic understanding of who is being stigmatized during the COVID-19 pandemic, what exacerbates COVID-19-related stigma, and what impacts COVID-19-related stigma has on victims. Therefore, this review aims to provide a systematic overview of COVID-19-related stigma. With 93 papers conducted with 126,371 individuals in more than 150 countries and territories spanning five continents, we identify three targets that have received the most research: Chinese/Asian people, (suspected) patients and survivors, and healthcare workers. Furthermore, we find that for each stigma target, characteristics of the stigmatized, stigmatizer, and context contribute to COVID-19-related stigma and that this stigma negatively influences victims' health and non-health outcomes. We call for future research to provide a more integrative, balanced, and rigorous picture of COVID-19-related stigma *via* conducting research on neglected topics (e.g., contextual factors that contribute to stigma toward HCWs) and stigma interventions and using a longitudinal design. In practice, we urge governments and institutions (e.g., ministries of public health, hospitals) to pay close attention to stigma issues and to promote safe and inclusive societies.

## Introduction

With more than 510 million cases and 6.23 million deaths worldwide (World Health Organization, 2022), COVID-19 is the largest human disaster since World War II and one of the greatest global public health threats in history (Qin et al., 2021; Wu J. et al., 2021; Robinson et al., 2022). This public health crisis has profound and unprecedented implications for societies and affects nearly every aspect of people's lives globally (Giuntella et al., 2021; Mckeown et al., 2021; Mueller et al., 2021). Besides its major health threats, what is equally important, if not more important in the long run, is the severe secondary impacts of COVID-19, one of which is the rise of social stigma. Social stigma refers to disapproval or discrimination of an individual based on some discrediting attributes that reduce a person from a normal and accepted person to a tainted and undesirable one (Goffman, 1963). Since the COVID-19 outbreak, social stigma has risen dramatically and directed to different targets, which we termed COVID-19-related stigma. For example, news and social media outlets have documented that anti-Asian hate crimes have surged (Timsit, 2022), survivors have been shunned by their friends and neighbors (Harmon, 2020), and healthcare workers have been denied access to community (Yeung and Gupta, 2020). On top of coping with the mounting stress over the spread of COVID-19 and its emerging new variants, stigmatized groups are additionally burdened by anxiety, aggression, and attacks (McKay et al., 2020). This stigma not only violates human rights but also threatens public health and impedes united efforts to combat the pandemic (World Health Organization, 2021).

Global leaders, organizations, and scholars have noticed this societal problem and have called upon the public to stop the stigmatization (Macias, 2020; Nature, 2020). For example, the United Nations International Children's Emergency Fund (UNICEF), the World Health Organization (WHO), and the International Federation of Red Cross and Red Crescent Societies (IFRC) published guidance on universal preventions to address the social stigma related to COVID-19 (UNICEF et al., 2020). Research has also been conducted to understand the detrimental consequences of COVID-19-related stigma and its potential antecedents. However, this documentation has been sporadic and scattered. This lack of a comprehensive understanding of COVID-19-related stigma impedes research on the salient but neglected aspects of the issue and hinders practical efforts to address this social problem. Thus, drawing on past research, we reviewed 93 papers that cover 126,371 individuals from more than 150 countries and territories spanning five continents to provide a holistic picture of COVID-19-related stigma. We aim to answer three questions in this review: Who are stigmatized during the pandemic? What contributes to the stigmatization of these people? What are the consequences for the stigmatized? This review is theoretically important because it synthesizes current findings and provides insights for future research on the COVID-19 stigma issue. Practically, this review could help decision makers in governments and institutions worldwide eliminate stigma and protect vulnerable groups.

Our review makes two primary contributions to the COVID-19 and stigma literature. First, we contribute to COVID-19 research by focusing on the social impacts of the pandemic. While the current literature largely focuses on the diagnostics, treatments, and vaccines for the disease (Älgå et al., 2020; Gupta et al., 2021), our review centers on stigmatized populations during the COVID-19 pandemic. Stigma can be seen as a social virus that could interfere with the containment of COVID-19 (Van Daalen et al., 2021) and may have a profound influence on those stigmatized even after the pandemic ends. By reviewing 93 papers on COVID-19-related stigma, we identify three groups as the main targets of COVID-19-related stigma—Chinese/Asian people, (suspected) patients and survivors, and HCWs—and provide an overview of these stigmatized groups during the pandemic. Second, for each target of COVID-19-related stigma, we review both the antecedents and outcomes of the stigma. Understanding the antecedents of COVID-19-related stigma is critical for reducing and preventing it as a deeper understanding will enable appropriate remedies for stigmatized groups, and knowledge about the outcomes of this stigma could raise concerns about its alarming effects and unite different social forces to combat this social issue.

In the following sections, we first report our literature review methodology. Next, we discuss the three main stigmatized targets that have emerged in the pandemic. Then, we summarize the antecedents and outcomes for each of the three COVID-19-related stigma targets. Finally, we discuss the findings and provide six suggestions for both research and institutions.

## Methods

This systematic review was conducted following the Preferred Reporting Items for Systematic Reviews (PRISMA) recommendations (Page et al., 2021). PRISMA is a comprehensive and most updated reporting protocol that ensures the quality of systematic reviews and is widely accepted in academic journals (e.g., Hammerstein et al., 2021; Lee et al., 2022). Based on PRISMA, we conducted the following processes.

### Identification of studies

We used Google Scholar to search for the following keywords: “stigma” or “discrimination” combined with “COVID-19” or “coronavirus.” We limited our search to articles published since 2020 because COVID-19 received attention from WHO on the last day of 2019 (World Health Organization, 2020), and we stopped our search on 31 December 2021. In addition, we used the backward reference searching method (i.e., examining the studies cited in the article) to identify potentially relevant studies from review articles on COVID-19-related stigma.

### Screening and selection of eligible studies

**Figure 1:**
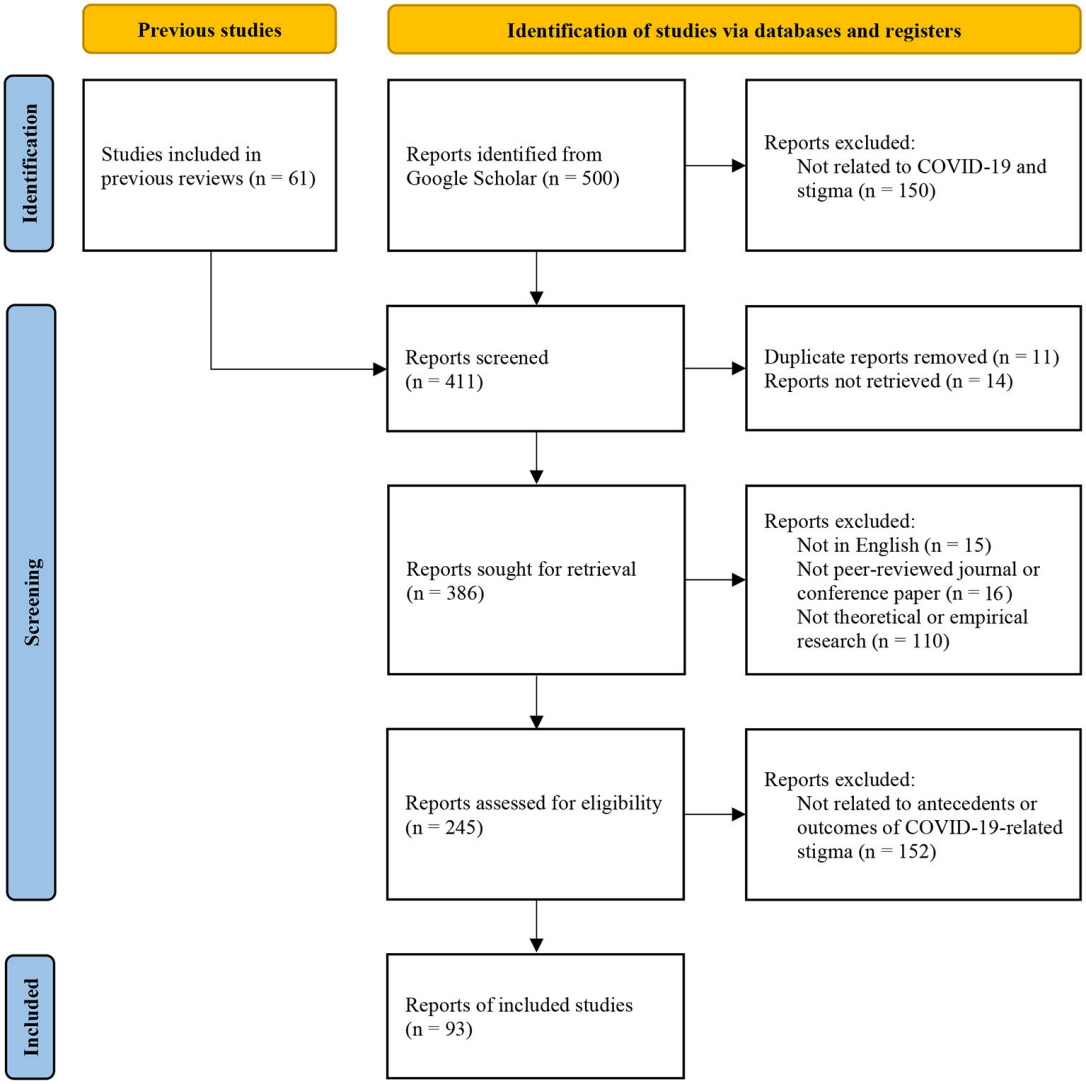
PRISMA.

The first, third, and fourth author independently screened the title and abstract of all studies from the initial searches to identify studies that could potentially be included. Then, the third and fourth author read the full text of the selected articles and assessed these articles against the full inclusion and exclusion criteria. Disagreements or ambiguities were resolved through discussion with all authors. The PRISMA diagram in Figure 1 depicts this process. To be included in our review, an article had to be (1) written in English, (2) be peer reviewed, (3) be an empirical or review paper, and (4) focus on antecedents or outcomes of COVID-19-related stigma. To reduce publication bias, we include research from difference sources such as peer reviewed journal and conference papers using Google Scholar database (Haddaway et al., 2015). In the end, 93 articles were included in our literature review (please see Supplementary material for the full list).

### Information extraction and coding

We used thematic analysis to synthesize the qualitative data we extracted from the articles (Braun and Clarke, 2006). We carried out three steps for the thematic analysis. First, the first, third, and fourth authors independently coded core topic investigated, antecedents, outcomes, and theory or mechanisms for each article and generated an initial list of codes for antecedents and outcomes for each COVID-19-related stigma targets. We also coded type of research (quantitative vs. qualitative), sample size, country information for each study. Second, the initial codes were combined into potential themes by the first author. Third, the author team reviewed, discussed, refined, and finalized the themes for this review.

## Targets of COVID-19-related stigma

**Figure 2:**
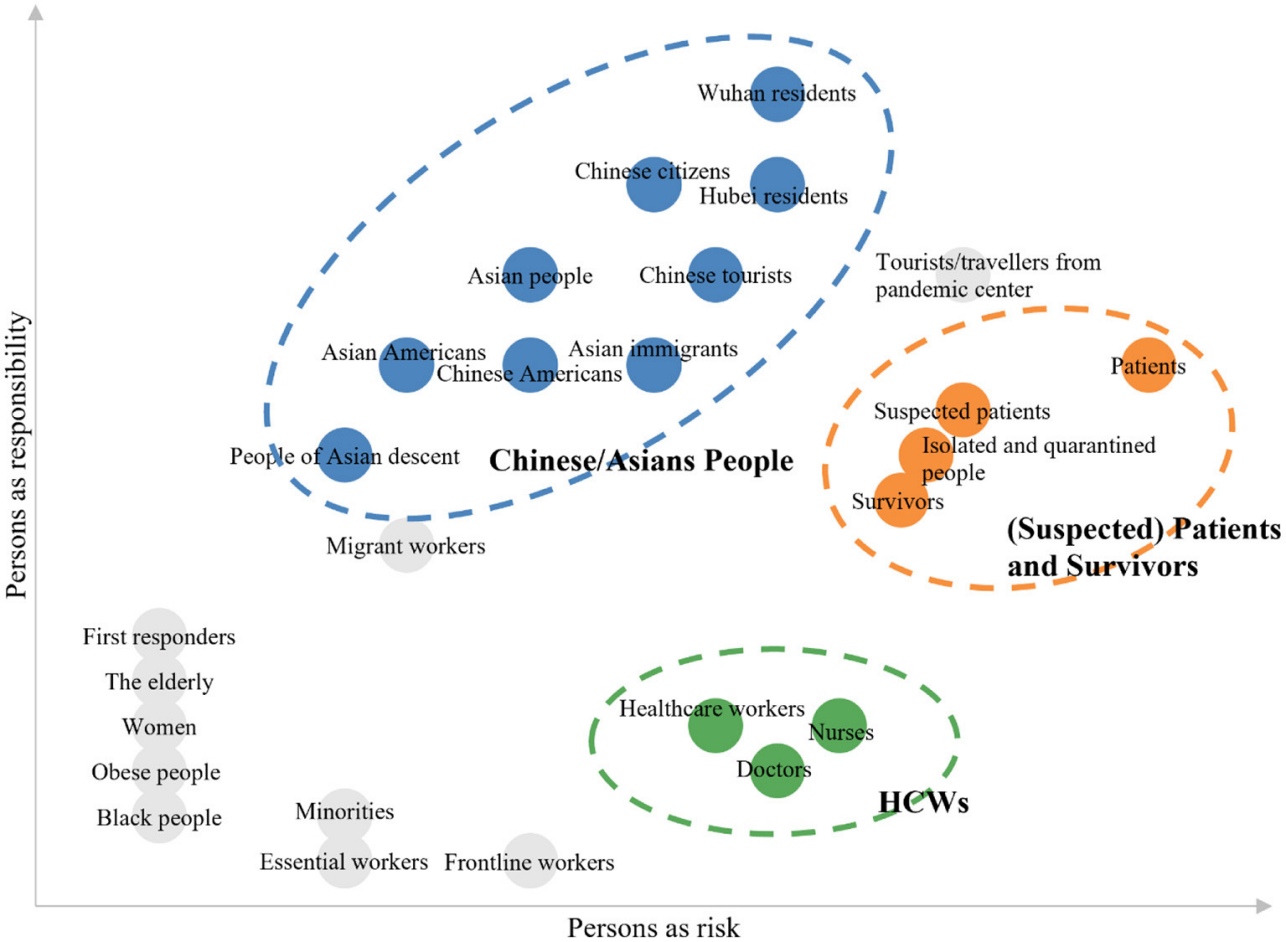
Summary of stigma targets during the COVID-19 pandemic.

Various victims of COVID-19-related stigma have been investigated in the literature. Figure 2 depicts all the stigma targets mentioned in the papers we initially reviewed. Following previous approach (Cho et al., 2021), we use two dimensions to categorize these targets: persons as risk (i.e., being perceived as posing risks to others) and persons as responsibility (i.e., being perceived as responsible for the risks). Stigma targets vary in these two dimensions. For example, at this stage where the infected cases are stably increasing, Chinese/Asian people may be perceived as high on persons as responsibility dimension and low on persons as risk dimension, while HCWs may be perceived as low on persons as responsibility dimension and medium on persons as risk dimension. In reviewing these articles, we identified three main groups of stigma targets that have received relatively more research attention in the literature: Chinese/Asian people, (suspected) patients and survivors, and HCWs.

### Stigma toward Chinese/Asian people

Chinese/Asian people have been frequently investigated as victims of stigma since the COVID-19 pandemic started. We include people from China or of Asian ancestry in this group, such as Wuhan residents, Hubei residents, Chinese citizens, Asian people, overseas Chinese and Asian people, and Asian immigrants. As Wuhan city in Hubei province, China, was the early epicenter of COVID-19 and is presumed to be the location of origin of the disease, Wuhan and Hubei residents, Chinese people, and Asian people have been particularly associated with COVID-19 and have thus become victims of stigma (Liu Y. et al., 2020; Xu et al., 2021).

Chinese/Asian people have encountered stigma at three levels during the COVID-19 pandemic. First, at the structural level, several government officials and institutions have referred to COVID-19 as the “Wuhan virus,” “Chinese Virus,” and “Kung Flu” even though alternative scientific names are available (Dhanani and Franz, 2020). Furthermore, stigma toward Chinese/Asian people has also been reinforced and perpetuated by social media (Duan et al., 2020). In addition, mainstream media outlets fueled stigma toward Chinese/Asian people because they frequently disseminated selective, biased, or incorrect information about COVID-19 (Dhanani and Franz, 2020). Second, at the interpersonal level, Chinese/Asian people have reported experiencing stigma and discrimination in different parts of the world, such as being accused of spreading disease, being treated with less courtesy, and being insulted or harassed [e.g., in France (Wang et al., 2020); in the United States (Yu et al., 2020; Lee and Waters, 2021); and in multiple countries (Ma and Miller, 2021)]. Third, at the intrapersonal level, Chinese/Asian people have internalized stigma and have reported feeling inferior (Fan et al., 2021).

### Stigma toward (suspected) patients and survivors

The second group of targets of COVID-19-related stigma we identified is (suspected) patients and survivors, including suspected and confirmed COVID-19 patients and people who have recovered from the disease. Many believe these people are at high risk of transmitting the disease, so they tend to be stigmatized after contracting COVID-19.

Stigma toward (suspected) patients and survivors is prevalent at the interpersonal and intrapersonal levels. First, COVID-19 patients suffer from verbal abuse, social isolation, and rejection (Atinga et al., 2021; Lin et al., 2021). These experiences of social stigma even spread to suspected patients (Liu Y. et al., 2020) and can persist into the recovery stage as numerous COVID-19 survivors have reported being excluded and rejected by neighbors, public spaces, workplaces, and healthcare facilities (Gopichandran and Subramaniam, 2021). Indeed, a large-scale study involving 1,212 participants from China showed that around one third of participants endorsed stigmatized attitude toward COVID-19 patients (Zhang et al., 2021). Second, at the intrapersonal level, COVID-19 patients' self-stigma manifests as feeling stressed about rejoining social activities and fear of being blamed for being outside (Lohiniva et al., 2021). COVID-19 survivors also experience internalized stigma, such as feeling like a bad person, and expected stigma, such as perceiving that people will reject them (Dar et al., 2020).

### Stigma toward HCWs

Despite being applauded and praised as our heroes in the media, HCWs have been stigmatized during the COVID-19 pandemic. This group of stigma targets includes nurses, doctors, and other HCWs (e.g., midwives). They are stigmatized due to their contact with COVID-19 patients and being perceived as potential carriers of the disease.

Most of the stigmatization toward HCWs happens at the interpersonal and intrapersonal levels. First, at the interpersonal level, doctors, nurses, and other HCWs have been accused of spreading the virus, denied essential services (e.g., shunned from grocery stores), ostracized, and even assaulted and attacked (Bhanot et al., 2021). Moreover, their family members often suffer from secondary, or associative, stigma and are harassed and bullied (Shiu et al., 2022). Second, at the intrapersonal level, studies have shown that HCWs experience internalized stigma, such as feeling stigmatized (Elhadi et al., 2020) and worrying about rejection and stigmatization (Greene et al., 2021).

**Figure 3:**
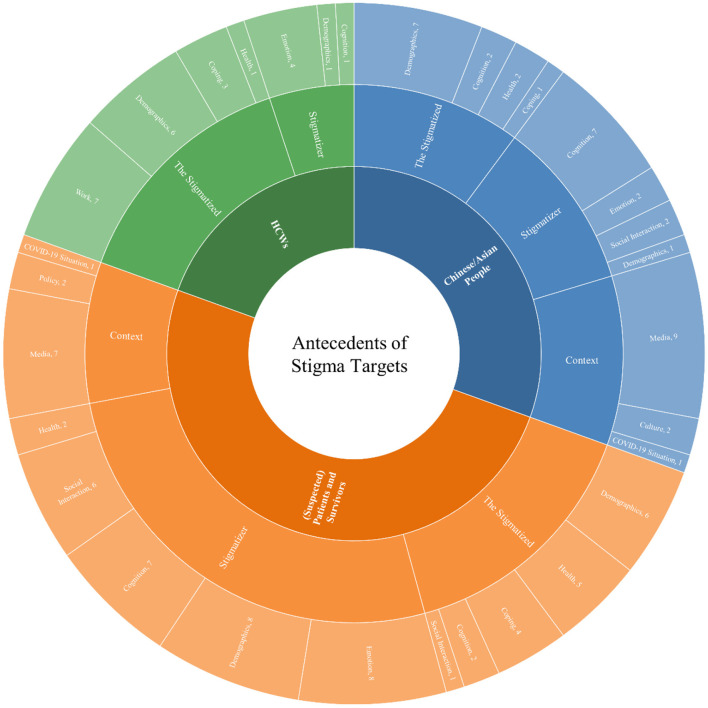
Summary of the antecedents for stigma targets.

**Figure 4:**
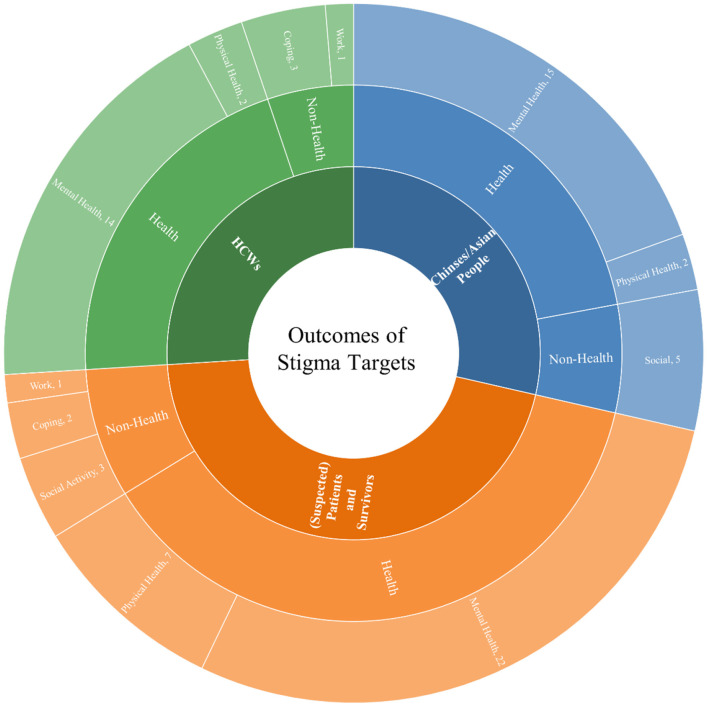
Summary of the outcomes for stigma targets.

In the following sections, we discuss the antecedents and outcomes associated with these three groups of stigma targets [i.e., Chinese/Asian people, (suspected) patients and survivors, and HCWs], respectively. We organize the antecedents of COVID-19-related stigma into three broad sections: the stigmatized, stigmatizer, and context. Figure 3 and Table 1 summarize the antecedents for each stigma target. The outcomes of COVID-19-related stigma are categorized as health- and non-health-related outcomes. Figure 4 and Table 2 summarize the outcomes for each stigma target.

**Table 1:** Antecedents for stigma targets.

**Stigma targets**	**Antecedent categories**	**Sub-categories**	**Specific variables**	**Sample countries**	**Representative research**
Chinese/Asian people	The Stigmatized	Demographics	Gender, age, education, occupation, SES, nationality, residential status, lived in Hubei, and Hubei *hukou* holders	Multiple countries, China, France, Jordan, United States	Chen X. et al., 2020; Duan et al., 2020; He et al., 2020; Wang et al., 2020; Alsawalqa, 2021; Fan et al., 2021; Litam and Oh, 2021
		Cognition	General: perceived relevance of stigmatized group	China	Chen X. et al., 2021
			COVID-19 related: perceived susceptibility to COVID-19, perceived severity of COVID-19, and familiarity with quarantined cases	China	Duan et al., 2020
		Health	General: Self-reported general health, anxiety symptoms	China	Duan et al., 2020
			COVID-19 related: confirmed or suspected to have COVID-19	China	Fan et al., 2021
		Coping	Face mask wearing	Poland	Rzymski and Nowicki, 2020
	Stigmatizer	Demographics	Gender, age, tenure, occupation, location of workplace, department, and marital status	Indonesia	Yufika et al., 2021
		Cognition	General: institutional trust, political trust, social trust, institutional efficacy, collective efficacy, self-efficacy, and Christian nationalism	Singapore, United States	Ahmed et al., 2021; Cho et al., 2021; Dhanani and Franz, 2020; Perry et al., 2021
			COVID-19 related: perceived magnitude of harm (severity) of COVID-19, COVID-19 risk perception, misconceptions about COVID-19, and knowledge of COVID-19	Indonesia, Singapore, South Africa, United States	Schmidt et al., 2020; Cho et al., 2021; Yufika et al., 2021
		Emotion	General: racial envy	United States	Cho et al., 2021
			COVID-19 related: fear, anger, and anxiety	Multiple counties, United States	Cho et al., 2021; Xu et al., 2021
		Social interaction (with the stigmatized group)	Social interaction (with Asian friends), parasocial interaction (with foreigners), and intergroup contact	United States	Tsai et al., 2020; Cho et al., 2021
	Context	Culture	Food culture, mask culture, racism, political ideology, openness to trade, and openness to migration	Multiple countries	He et al., 2021; Xu et al., 2021
		Media	Media use, social media use, news consumption, media exposure, media engagement, media trust, framing, media sources, social network size, and social network heterogeneity	China, Singapore, United States	Croucher et al., 2020; Dhanani and Franz, 2020, 2021; Duan et al., 2020; Tsai et al., 2020; Yu et al., 2020; Ahmed et al., 2021; Cho et al., 2021; Haft and Zhou, 2021
		COVID-19 situation	Infected number in the country	Multiple countries	He et al., 2020
(Suspected) patients and survivors	The Stigmatized	Demographics	Gender, age, education, occupation, employment status, income, financial situation, marital status, race, immigrant status, residence, and family infection history	Canada, China, India, Saudi Arabia, United States	Dar et al., 2020; Al-Zamel et al., 2021; Lin et al., 2021; Miconi et al., 2021b; Yuan et al., 2021
		Cognition	COVID-19 related: blaming self for contracting the disease, being blamed for contacting coronavirus, uncertainty about immunity, and uncertainty about having contacted coronavirus	Multiple countries, Finland	Ransing et al., 2020; Lohiniva et al., 2021
		Health	COVID-19 related: Symptoms continue after quarantine, severity of disease, perceived health status, presence of comorbidities, and time since discharge (days)	China, Finland, India, Saudi Arabia	Dar et al., 2020; Al-Zamel et al., 2021; Lin et al., 2021
		Coping	Quarantine status, COVID-19 status (isolated vs. quarantined), wear a face mask, and use of personal protective equipment	Bahrain, China, United States	Liu Y. et al., 2020; Nursalam et al., 2020; Xin et al., 2020; Jassim et al., 2021
		Social interaction	Social activities	United States	Liu Y. et al., 2020
	Stigmatizer	Demographics	Gender, age, education, occupation, employment status, income, marital status, ethnicity, residence, region, and working province	Bangladesh, China, Columbia, Jordan, Nepal	Abuhammad et al., 2021; Cassiani-Miranda et al., 2021; Hossain et al., 2021; Jiang et al., 2021; Li et al., 2021; Singh et al., 2021; Zhang et al., 2021
		Cognition	General: feeling of resource scarcity, trust in public officials, trust in health experts, trust in general public, cognitive load, and need to belong	China	Chen et al., 2021a
			COVID-19 related: misbeliefs, negative attitudes toward COVID-19, knowledge of COVID-19, attribution of COVID-19, risk perception of COVID-19, vaccine effectiveness, vaccine intention, and objectification (of people returning from Hubei)	Multiple countries, Bangladesh, China, Lebanon	Ransing et al., 2020; Hossain et al., 2021; Li et al., 2021; Zhang et al., 2021
		Emotion	General: optimism	China	Chen et al., 2021a
			COVID-19 related: fear, terror, COVID-19 anxiety, anger toward COVID-19 patients, dangerousness of COVID-19 patients, worry, and feeling of vulnerability	Multiple countries, Colombia, China, India, Lebanon, Russia	Ransing et al., 2020; Sorokin et al., 2020; Cassiani-Miranda et al., 2021; Chen et al., 2021a; Gopichandran and Subramaniam, 2021; Haddad et al., 2021; Zhang et al., 2021
		Health	General: Self-rated health	China	Li et al., 2021
			COVID-19 related: Own COVID-19 status	Bangladesh	Hossain et al., 2021
		Social interaction (with the stigmatized group)	Infections of relatives and friends, served COVID-19 patients, history of COVID-19 in the family, direct or indirect contact with a (suspected) COVID-19 individual, and know someone who was COVID-19 positive within their immediate social environment, and epidemic proximity	Bangladesh, China, Iraq, Lebanon, Nepal	Chen et al., 2021a; Haddad et al., 2021; Hossain et al., 2021; Li et al., 2021; Singh et al., 2021
	Context	Media	Infodemics, social media use, fake news, media quality, concealment of information, lack of transparency, and irresponsible media coverage	Multiple countries, China, India, Sri Lanka, United States	Liu Y. et al., 2020; Nursalam et al., 2020; Ransing et al., 2020; Gopichandran and Subramaniam, 2021; Jayakody et al., 2021; Sahoo and Patel, 2021; Yuan et al., 2021
		Policy	Involvement of police, forced isolation, and different and conflicting information about COVID-19 and quarantine procedures	Finland, India	Gopichandran and Subramaniam, 2021; Lohiniva et al., 2021
		COVID-19 situation	Province by confirmed patients	China	Jiang et al., 2021
HCWs	The Stigmatized	Demographics	Gender, age, education, tenure, marital status, residence, province, and living with the elderly	Bangladesh, India, Iran, Nepal	Yadav et al., 2020; Zandifar et al., 2020; Adhikari et al., 2021; Radhakrishnan et al., 2021; Uvais et al., 2021; Khan et al., 2022
		Work	Frontline worker, designation, department, occupation, treating COVID-19 patients, and type of healthcare facility	Bangladesh, Colombia, India, Iran, Nepal	Yadav et al., 2020; Adhikari et al., 2021; Radhakrishnan et al., 2021; Khan et al., 2022
		Health	COVID-19 diagnosed, chronic diseases, history of psychiatric illness, and admitted to the hospital due to COVID-19	Nepal	Adhikari et al., 2021
		Coping	Precautionary measures, being in quarantine, and history of quarantine	Bangladesh, India, Nepal	Adhikari et al., 2021; Uvais et al., 2021; Khan et al., 2022
	Stigmatizer	Demographics	Gender, education, occupation, employment status, income, marital status, and have children	Columbia	Cassiani-Miranda et al., 2021
		Cognition	General: feeling of resource scarcity, trust in public officials, trust in health experts, trust in general public, cognitive load, and need to belong	China	Chen et al., 2021a
			COVID-19 related: (COVID-19) knowledge acquisition, (COVID-19) knowledge seeking effort, and objectification (of people returning from Hubei)	China	Chen et al., 2021a
		Emotion	General: optimism	China	Chen et al., 2021a
			COVID-19 related: fear, COVID stress, worry, and feeling of vulnerability	Canada, Columbia, China, United States, Canada	Taylor et al., 2020; Cassiani-Miranda et al., 2021; Chen et al., 2021a

**Table 2:** Outcomes for stigma targets.

**Stigma targets**	**Outcome categories**	**Sub-categories**	**Specific variables**	**Sample countries**	**Representative research**
Chinese/Asian people	Health	Mental health	Depression, anxiety, fear, mental disorder, psychological distress, psychological well-being, anger, grief, internalizing and externalizing problems, life satisfaction, PTSD, and self-esteem	Multiple countries, China, Jordan, United States	Cheah et al., 2020; Litam and Oh, 2020, 2021; Yang and Wong, 2020; Alsawalqa, 2021; Chen X. et al., 2021; Chen et al., 2021b; Fan et al., 2021; Haft and Zhou, 2021; Hahm et al., 2021a,b; Lee and Waters, 2021; Ma and Miller, 2021; Maglalang et al., 2021; Wu C. et al., 2021
		Physical health	Physical symptoms, self-rated health, and sleep difficulties	Multiple countries, United States	Lee and Waters, 2021
	Non-health	Social activity	Social media use, avoidance, internalizing difficulties, social support, and altruistic tendency	Multiple countries, China, United States	Yang et al., 2020; Chen et al., 2021b; Chen X. et al., 2021; Hahm et al., 2021b
(Suspected) patients and survivors	Health	Mental health	Mental health, mental well-being, well-being, depression, suicide, self-harm, anxiety, emotional distress, psychological distress, PTSD, feeling of loneliness, feeling of uncertainty, feeling of helplessness, quality of life, worry, guilt, and fear	Multiple countries, Bangladesh, Canada, China, Finland, Ghana, India, Indonesia, Iran, Istanbul, Italy, Korea, Saudi Arabia, Sri Lanka, United States	Liu D. et al., 2020; Ransing et al., 2020; Xin et al., 2020; Adom et al., 2021; Al Eid et al., 2021; Atinga et al., 2021; Gopichandran and Subramaniam, 2021; Grover et al., 2021; Harjana et al., 2021; Jayakody et al., 2021; Kang et al., 2021; Mahmoudi et al., 2021; Miconi et al., 2021a; Paleari et al., 2021; Poyraz et al., 2021; Sahoo and Patel, 2021; Yuan et al., 2021; Gan et al., 2022
		Physical health	Delay in recovery, persistence of symptoms, insomnia, eating disorder, physical quality of life, COVID-19 testing, reluctance to report symptoms and seek treatment, concealment of contact with COVID-19 patients, and reduced capacity of the healthcare system	Multiple countries, China, Finland, Ghana, India, Iran, Istanbul, United States	Earnshaw et al., 2020; Ransing et al., 2020; Atinga et al., 2021; Bhanot et al., 2021; Lohiniva et al., 2021; Mahmoudi et al., 2021; Poyraz et al., 2021
	Non-health	Social activity	Social contact, anthropophobia, and social isolation	Finland, Ghana, India	Atinga et al., 2021; Lohiniva et al., 2021
		Work	Poor motivation, and compromised engagement in employment	Multiple countries	Ransing et al., 2020
		Coping	Prolonged quarantine time, reduced compliance with self-isolation rules and guidelines, and coping mechanisms (including disclosing infection, following quarantine instructions, and convincing self)	Finland	Saeed et al., 2020; Lohiniva et al., 2021
HCWs	Health	Mental health	Anxiety, depression, PTSD, mental health, psychological well-being, stress, psychological distress, burnout, negative emotions, suicide risk, and resilience	Bangladesh, Columbia, China, India, Iran, Libya, Nepal, Philippines, Singapore, Turkey, United Kingdom	Elhadi et al., 2020; Khanal et al., 2020; Teksin et al., 2020; Campo-Arias et al., 2021; Greene et al., 2021; Kirk et al., 2021; Patel et al., 2021; Shiu et al., 2022
		Physical health	Physical quality of life and insomnia	Nepal, Turkey	Khanal et al., 2020; Teksin et al., 2020
	Non-health	Work	Turnover intention	Philippines	Labrague et al., 2021
		Coping	Alcohol use	United States	Zolnikov and Furio, 2020

## Antecedents of stigma toward Chinese/Asian people

For each target of COVID-19-related stigma, we organize the antecedents into three broad categories: the stigmatized, stigmatizer, and context.

### The stigmatized

Previous studies have examined how characteristics of the stigmatized (i.e., Chinese/Asian people) affect their perceived stigma. We organize these studies into five sections: demographics, cognition, health, coping, and social interaction.

#### Demographics

The demographics of the stigmatized can influence perceived stigma in two ways (Chen X. et al., 2020; Duan et al., 2020; He et al., 2020; Alsawalqa, 2021; Fan et al., 2021; Litam and Oh, 2021): (1) some demographics (e.g., Hubei residence, and Hubei *hukou*, which refers to households registered in Hubei province) increase Chinese/Asian people's relevance to the pandemic, leading to increased perceived stigma, and (2) Chinese/Asian people with certain demographics [e.g., male, young age, and low socioeconomic status (SES)] are more sensitive to stigma and/or less capable of coping with stigma, resulting in higher perceived stigma.

First, Chinese/Asian people with certain demographics (e.g., Hubei residence, and Hubei *hukou*) are seen as more relevant to the pandemic, resulting in more stigma toward these people. For example, one study found that because Hubei had a large proportion of cases in the early stage of the COVID-19 pandemic and Hubei people were accused of moral transgressions (e.g., eating wild animals), people who lived in Hubei during China's first COVID-19 outbreak and Hubei *hukou* holders faced higher discrimination (Fan et al., 2021). Similarly, one study found that compared to students from other East and Southeast Asian countries (e.g., Korea and Singapore), Chinese students have suffered significantly more cyberbullying (Alsawalqa, 2021).

Second, Chinese/Asian people with certain demographics (e.g., male, young age, and low SES) are more sensitive to others' stigmatizing behavior and/or less capable of coping with stigma, so they perceive higher levels of stigma. For example, Chinese/Asian men are more likely to report stigma experiences (Chen X. et al., 2020; Litam and Oh, 2021) because they tended to experience less stigma before the pandemic, making their experiences of COVID-19-related stigma more salient. Chinese/Asian women, on the other hand, may attribute stigma to their gender and thus tend to report less COVID-19-related stigma (Litam and Oh, 2021). Young Chinese/Asian people, who are less capable of coping with stigma compared to older people, tend to report higher stigma (Chen X. et al., 2020; He et al., 2020; Wang et al., 2020). Similarly, Hubei residents with low SES have fewer resources to protect their social image and have been found to report more stigma (Duan et al., 2020). However, the results on education are rather mixed. While one study found that education is positively related to stigma (Duan et al., 2020), one study found a null relationship (Chen X. et al., 2020) and another study found a negative relationship (He et al., 2020).

#### Cognition

Two cognition-related aspects can influence Chinese/Asian people's perceived stigma: general and COVID-19-related cognition. Specifically, general cognition [e.g., perceived relevance of the stigmatized (Chen X. et al., 2021)] affects Chinese/Asian people's perceived stigma mainly because Chinese/Asian people's perceived relevance of the stigmatized increases their identification with a stigmatized group and their sensitivity to stigma posed by external groups, which increases their perceived stigma. COVID-19-related cognition [e.g., perceived higher susceptibility/severity of COVID-19 (Duan et al., 2020)] affects Chinese/Asian people's perceived stigma mainly *via* aggravating their anticipated stigma.

For general cognition, Chinese/Asian people who perceive higher relevance of the stigmatized group tend to identify more strongly with the group and are more sensitive to the stigma from external groups, which is related to increased perceived stigma. Indeed, one study found that perceived higher relevance of the stigmatized group is associated with higher levels of perceived stigma (Chen X. et al., 2021).

For COVID-19-related cognition, Chinese/Asian people who feel amplified perceived threats of COVID-19 may anticipate more stigma toward themselves, which is associated with increased stigma perceptions. For example, one study found that Chinese people who perceive higher susceptibility or severity of COVID-19 tend to report higher levels of perceived stigma (Duan et al., 2020).

#### Health

Two health-related aspects can influence Chinese/Asian people's perceived stigma: general and COVID-19-related health. Specifically, general health (e.g., anxiety disorder symptoms) may influence Chinese/Asian people's perceived stigma because it may indicate amplified risk perceptions. COVID-19-related health (e.g., being suspected or confirmed of having COVID-19) increases Chinese/Asian people's stigma perceptions because being a suspected or confirmed COVID-19 patient brings additional stigma [i.e., stigma toward (suspected) patients].

For general health, Chinese/Asian people's anxiety symptoms may indicate that they have amplified risk perceptions of themselves transmitting the disease, resulting in higher stigma perceptions. For example, one study found that Chinese people who report more anxiety disorder symptoms (rather than self-reported general health) are more likely to report higher levels of perceived stigma (Duan et al., 2020).

For COVID-19-related health, Chinese/Asian people who are confirmed or suspected COVID-19 patients perceive more stigma due to having two stigmatized identities (i.e., being Chinese/Asian and being COVID-19 patients). For example, in a sample of 7,942 Chinese people, one study found that being confirmed or suspected of having COVID-19 is significantly related to perceived discrimination regardless of whether control variables, such as gender, age, education, income etc., are included or excluded (Fan et al., 2021).

#### Coping

Chinese/Asian people's use of coping strategies, such as face mask wearing (Rzymski and Nowicki, 2020), increases their stigma perceptions because of public misassumptions that wearing masks means there is a threat of COVID-19. For example, one study found that Asian medical students who wear face masks more frequently experience prejudice related to the COVID-19 outbreak in Poland compared to Asians who do not wear masks (Rzymski and Nowicki, 2020).

### Stigmatizer

Based on studies we reviewed, stigmatizers' influence on stigma toward Chinese/Asian people can be organized into four sections: demographics, cognition, emotion, and social interaction.

#### Demographics

Stigmatizers' demographics (e.g., occupation, and workplace characteristics) influence their stigma attitudes toward Chinese/Asian people because certain demographics reflect stigmatizers' low knowledge and/or high perceived risk of COVID-19 (Yufika et al., 2021). For example, one study found that compared with doctors, nurses are more likely to display stigma toward Chinese/Asian people because they have lower knowledge and perceive higher risk of COVID-19 (Yufika et al., 2021). As another example, HCWs who work in private sub-rural hospitals and hospitals with no protocols for triage and isolation for patients may perceive higher risk and have lower knowledge about COVID-19, and they have been found to have more stigmatizing attitudes toward Chinese/Asian people (Yufika et al., 2021). However, no significant relationships have been found between stigma and stigmatizers' other demographics (e.g., gender, age, and marital status).

#### Cognition

Stigmatizers' cognition, both general and COVID-19 related, affects their stigmatizing attitudes and behavior. Specifically, stigmatizers' general cognition affects stigmatizing attitudes and behavior in two ways: (1) stigmatizers' sense of efficacy and trust toward the collective and institutions (e.g., social/political trust, and collective efficacy) enhance their positive appraisals of resources to cope with COVID-19, which mitigates stigmatizing behavior, and (2) positive attitudes toward Trump's government [e.g., trust in Trump (Dhanani and Franz, 2020)] are associated with stigmatizing attitudes toward Chinese/Asian people following former President Trump's stigmatizing behavior (e.g., repeatedly using “Chinese virus” to refer to COVID-19). COVID-19-related cognition affects stigmatizers' stigmatizing behavior mainly because individuals with correct knowledge and perceptions about COVID-19 engage in less stigmatizing behavior.

For general cognition, first, people who perceive the collective or institutions as credible and efficacious (e.g., social/political trust, and collective efficacy) have more positive appraisals of their resources to cope with COVID-19; therefore, they are less likely to stigmatize Chinese/Asian people. For example, one study found that Singaporean citizens with higher trust in the government and people in society are less likely to adopt stereotypes and prejudice against Chinese immigrants (Ahmed et al., 2021). Another study found that collective efficacy, but not self-efficacy, is negatively related to stigmatizing attitudes toward Chinese/Asian people (Cho et al., 2021).

Second, given former President Trump's frequent stigmatizing behavior toward Chinese people (e.g., using “Chinese virus” to refer to COVID-19), stigmatizers who have more positive attitudes toward him and his government are more likely to engage in stigmatizing behavior toward Chinese/Asian people. For example, one study found that trust in Trump is significantly related to negative attitudes toward Asian Americans in response to COVID-19 (Dhanani and Franz, 2020). Another study found that perceived institutional efficacy (in Trump's government) during COVID-19 is positively related to stigmatizing attitudes toward Chinese/Asian people (Cho et al., 2021). In addition, Christian nationalism, “one of the leading factors driving continued support for Donald Trump and his policies” (Perry et al., 2021, p. 760), is significantly associated with supporting stigmatizing opinions about Chinese/Asian people (Perry et al., 2021).

For COVID-19-related cognition, individuals with high levels of knowledge and correct perceptions about COVID-19 are more likely to have low levels of stigmatizing attitudes and behavior. For example, one study found that HCWs with higher knowledge about COVID-19 are less likely to have stigmatizing attitudes toward Chinese/Asian people (Yufika et al., 2021). One study conducted a qualitative study in South Africa and found that misconceptions about COVID-19 are prevalent and contributes to stigmatizing attitudes and behavior toward people of Asian descent (Schmidt et al., 2020). Another study found that the perceived magnitude of harm of COVID-19 is positively associated with stigmatizing attitudes toward Chinese/Asian people (Cho et al., 2021).

#### Emotion

Stigmatizers' negative emotions, both general and COVID-19 related, affect their stigmatizing behavior. Specifically, general emotions [e.g., racial envy (Cho et al., 2021)] signal prejudice of Chinese/Asian people, which is positively related to stigmatizing attitudes toward this group. COVID-19-related emotions [e.g., fear of infection (Xu et al., 2021)] are also positively related to stigmatizing attitudes toward Chinese/Asian people because stigmatization is used as a coping mechanism for negative emotions arising from the pandemic.

For general emotions, racial envy signals prejudice against Chinese/Asian people and perceived ineligibility of their advantages over other races, which may promote harm and stigma toward Chinese/Asian people. For example, one study found that American adults' racial envy is positively related to stigmatizing attitudes toward Chinese/Asian people (Cho et al., 2021).

For COVID-19-related emotions, negative emotions are positively related to stigmatizing attitudes toward Chinses/Asian people as many use stigmatization as a coping mechanism against negative emotions arising from the pandemic (e.g., fear). For example, one study found that COVID-19 creates public panic due to uncertainty about COVID-19 infections, resulting in avoidance and stigmatization of people believed to be at high risk of spreading the virus, which were mainly Chinese people at the onset of the pandemic (Xu et al., 2021). In addition, one study found that fear, rather than anger and anxiety, is positively related to stigmatizing attitudes toward Chinese/Asian people (Cho et al., 2021).

#### Social interaction (with the stigmatized group)

Stigmatizers' social interaction with the stigmatized group [e.g., social interaction with Asian friends (Cho et al., 2021), and intergroup contacts (Tsai et al., 2020)] could influence their stigmatizing attitudes in two contrasting ways: (1) interacting with Chinese/Asian people heightens the risk of infection, which is associated with more stigmatizing attitudes, (2) interacting with Chinese/Asian people can enhance understanding of this group and thus reduce stigma.

First, social interaction with Chinese/Asian people activates the threat and risk of COVID-19 infection, which is associated with more stigmatizing attitudes. For example, one study found that the frequency of direct intergroup contact is positively associated with prejudicial attitudes toward Asian people because of higher perceived threats (Tsai et al., 2020).

Second, interaction with Chinese/Asian people can enhance understanding of this group, thus stigmatizers are more likely to change their beliefs and stigmatizing attitudes toward Chinese/Asian people after interacting with them. For example, one study found that parasocial interaction with foreigners (i.e., watching foreign language movies) is negatively related to stigmatizing attitudes toward Chinese/Asian people (Cho et al., 2021). However, direct social interaction (i.e., number of close Asian friends) is not significantly related to stigmatizing attitudes.

### Context

In terms of contextual antecedents for stigma toward Chinese/Asian people, we organize these studies into three sections: culture, media, and COVID-19 situation.

#### Culture

Culture [e.g., food culture, mask culture, and racism (Xu et al., 2021)] influences stigma toward Chinese/Asian people in two primary ways: (1) food/mask culture contributes to stigmatizing behavior toward Chinese/Asian people because Westerners vs. Chinese/Asian people interpret food/mask wearing differently, and (2) deeply rooted racism toward Chinese/Asian people and political tension between mainland China and Hong Kong/Taiwan have been revived and intensified during the COVID-19 pandemic, which has also increased stigma toward Chinese/Asian people.

First, food/mask culture contributes to stigma toward Chinese/Asian people because Westerners vs. Chinese/Asians have different interpretations of food and mask wearing. Many people from Western countries consider eating wild animals uncivilized and dirty and believe that Wuhan people's consumption of wild animals caused the virus (Xu et al., 2021). Additionally, while overseas Chinese people wear masks for self-protection, people from Western countries may mistake this practice as spreading COVID-19 or creating terror and panic. For example, one study summarized that differences in food/mask culture comprise one of the factors contributing to stigma toward Wuhan residents and overseas Chinese/Asian people (Xu et al., 2021).

Second, racism against Chinese/Asian people has a long history in United States (Tessler et al., 2020), and political tension between mainland China and Hong Kong/Taiwan has been a longstanding issue (Xu et al., 2021). Both of these issues have been revived and intensified during the COVID-19 pandemic and contribute to stigma toward Chinese/Asian people. For example, one study found that racism and hate crimes toward Chinese/Asian people have been widely reported in multiple media outlets (e.g., CNN, *New York Times*) and that former US President Trump's use of stigmatizing words (e.g., “Chinese virus”) fueled racial discrimination (Xu et al., 2021). In addition, mainland China and Hong Kong/Taiwan have different political ideologies and have handled COVID-19 differently. As such, COVID-19 has heightened the political tension between the two regions and has fueled discrimination and stigma toward mainland Chinese people (Xu et al., 2021). In contrast, country-level openness to trade and immigration (measured by the value of trade flow relative to national GDP, and immigration flow relative to population, respectively), promote interaction between people of different races and nationalities, which has increased mutual understanding and decreased stigma toward Chinese/Asian people. For example, a multi-country study found that more openness to trade and immigration with China was related to reduced reported discrimination (He et al., 2021).

#### Media

Media factors [e.g., (social) media use (Croucher et al., 2020; Dhanani and Franz, 2020; Duan et al., 2020; Tsai et al., 2020; Yu et al., 2020; Ahmed et al., 2021; Cho et al., 2021; Haft and Zhou, 2021), media sources (Tsai et al., 2020), framing (Dhanani and Franz, 2021)] have been extensively investigated in the literature. Three major points highlight the media's influence on stigma toward Chinese/Asian people: (1) people who use certain types of media (e.g., right-leaning media, and social media) are more likely to stigmatize Chinese/Asian people because biased and fake information is prevalent on these media platforms, (2) Chinese/Asian people who use media more frequently are more likely to perceive stigma toward themselves due to exposure to the biased and negative information in social and traditional media, and (3) the algorithms of media platforms may reinforce biased information and beliefs.

First, according to cultivation theory, right-leaning traditional media outlets politicize COVID-19 and blame China for the pandemic, aggravating the use of stigmatizing words, such as “China virus” and “Wuhan virus” (Tsai et al., 2020). Additionally, due to the lack of supervision of social media, users can freely publish information, resulting in substantial biased and false information spreading rapidly and widely on the Internet (Dhanani and Franz, 2020; Cho et al., 2021). Therefore, when individuals use right-leaning and social media more frequently, they tend to receive more biased and inaccurate information and form prejudicial and stereotypical attitudes, resulting in more stigma toward Chinese/Asian people. For example, one study found that use of traditional media and trust in social media are positively associated with prejudicial attitudes toward Asian people and relying on left-leaning and neutral media is negatively related to prejudicial attitudes toward Asian people (Tsai et al., 2020). Trust in social media also reinforces the effect of social media use on prejudice against Asian people such that frequent users of social media are more likely to have prejudicial attitudes toward Asian people when they have more trust in social media. In addition, how media outlets frame COVID-19 affects users' stigmatizing attitudes. For example, one study conducted an experiment and found that using Chinese framing (vs. neural framing) when describing the origin of COVID-19 significantly contributes to stigmatizing attitudes toward Asian people (Dhanani and Franz, 2021).

Second, Chinese/Asian people who use media more frequently are more likely to be exposed to biased information, which may result in higher perceived stigma toward themselves. For example, one study found that Hubei people who use media more frequently are more likely to report stigma (Duan et al., 2020). One study found that exposure to negative media portrayals of Chinese people mediates the relationship between COVID-19 period (i.e., pre- vs. post-COVID-19) and perceived discrimination among Chinese American college students (Haft and Zhou, 2021). Another study also found that use of social media is positively related to both experiences of everyday discrimination and concerns about future discrimination in a sample of Asians and Asian Americans (Yu et al., 2020).

Third, the algorithms of social media platforms can make individuals receive information they tend to agree with, which would create an echo chamber effect, leading to selective attention and polarization of opinions (Croucher et al., 2020; Ahmed et al., 2021). As such, social media may further worsen the problem of stigma toward Chinese/Asian people. For example, one study found that participants who use Facebook, a social media platform that uses algorithms to create a newsfeed (Bucher, 2017), score significantly higher on prejudice against Asian people (Dhanani and Franz, 2020). To mitigate the echo chamber effect created by algorithms, one study suggested increasing network size and heterogeneity, as they found that large network size and discussion network heterogeneity are negatively related to stereotypes and prejudice against Chinese immigrants (Ahmed et al., 2021).

#### COVID-19 situation

The COVID-19 situation [e.g., number of infected individuals in a country (He et al., 2020)] has been investigated as an influence on stigma behavior toward Chinese/Asian people because when COVID-19 is more prevalent in a country, people may perceive less health risk and are less likely to engage in stigmatizing behavior (Robinson and Daly, 2021). Indeed, one study found that overseas Chinese people living in countries with more confirmed cases are less likely to experience discrimination and violent overaction (He et al., 2020).

## Antecedents of stigma toward (suspected) patients and survivors

### The stigmatized

Studies have examined how characteristics of the stigmatized [i.e., (suspected) patients and survivors] affect their perceived stigma, and we categorize these studies into four sections: demographics, cognition, health, and coping.

#### Demographics

The demographics of (suspected) patients and survivors have been investigated in the literature (Dar et al., 2020; Al-Zamel et al., 2021; Lin et al., 2021; Miconi et al., 2021b; Yuan et al., 2021). The influence of (suspected) patients and survivors' demographics can be interpreted in two main ways: (1) (suspected) patients and survivors whose demographics indicate a lack of resources are more vulnerable (e.g., economic loss) and tend to report higher stigma, and (2) (suspected) patients and survivors with certain demographics (e.g., men) tend to perceive higher stigma because they experienced less stigma before the pandemic and are more sensitive than their counterparts.

First, (suspected) patients and survivors whose demographics indicate a lack of resources (e.g., economic loss) are more vulnerable and more susceptible to the negative influence of stigma. Thus, they may perceive higher stigma. For example, one study found that COVID-19 survivors who have faced higher economic losses perceive higher levels of stigma (Yuan et al., 2021).

Second, (suspected) patients and survivors with certain demographics (e.g., men) had fewer stigmatizing experiences before the pandemic and are more sensitive to stigma toward them, so they tend to report higher stigma perceptions than female (suspected) patients and survivors. Indeed, two studies found that male (suspected) patients and survivors had higher perceived stigma (Dar et al., 2020; Miconi et al., 2021b).

#### Cognition

(Suspected) patients and survivors' COVID-19-related cognition impacts their perceived stigma in two ways: (1) (suspected) patients and survivors who blame themselves or perceive being blamed for contracting COVID-19 may anticipate more stigma, which contributes to their stigma perceptions, and (2) (suspected) patients and survivors who are uncertain about their COVID-19 status may feel negative about themselves, contributing to self-stigma.

First, (suspected) patients and survivors who blame themselves or perceive being blamed for contracting COVID-19 may anticipate more stigma toward themselves, resulting in increased stigma perceptions. For example, a qualitative study in Finland showed that one of the drivers of COVID-19 patients' perceived stigma is being blamed for contracting the virus (Lohiniva et al., 2021). In addition, a review paper suggested that self-blame could be an important contributor of stigma perceptions (Ransing et al., 2020).

Second, (suspected) patients and survivors who are uncertain about their COVID-19 status may have negative feelings toward themselves, resulting in self-stigma. For example, one study found that uncertainty about their COVID-19 status and their immunity contributes to suspected and confirmed patients' self-stigma during and even after quarantine (Lohiniva et al., 2021).

#### Health

(Suspected) patients and survivors' health conditions [e.g., severity of disease (Lin et al., 2021), time since hospital discharge (Dar et al., 2020)] influence their stigma perceptions because their poor health conditions may imply higher risk of transmitting the disease, resulting in more perceived stigma. For example, one study found that compared with patients with mild symptoms, COVID-19 patients whose condition was more severe perceive higher stigma (Lin et al., 2021). In addition, one study found that with increasing time since hospital discharge, survivors' self-reported stigma decreases (Dar et al., 2020). However, another study found that the presence of comorbidities is not related to perceived stigma (Al-Zamel et al., 2021).

#### Coping

(Suspected) patients and survivors' use of coping strategies [e.g., wearing a face mask and other personal protective equipment (Liu Y. et al., 2020; Nursalam et al., 2020), being quarantined (Nursalam et al., 2020; Xin et al., 2020; Jassim et al., 2021)] signals heightened risk of transmitting COVID-19, resulting in more perceived stigma. Specifically, quarantine and mask wearing send a risk signal that people who are quarantined or wear face masks are more likely to carry the virus (Liu Y. et al., 2020; Xin et al., 2020; Jassim et al., 2021). Thus, (suspected) patients and survivors using these coping strategies are more likely to be stigmatized. For example, one study found that being quarantined positively predicts perceived discrimination because of presumed COVID-19 infection (Xin et al., 2020). One study also found that individuals who wear face masks experience more stigma because of suspected COVID-19 status than those who do not (Liu Y. et al., 2020). Furthermore, the study found that mask wearing interacts with working status to predict experienced stigma such that those working outside and wearing masks experience more stigma compared with those who do not wear masks and those who wear masks and work partially or fully from home (Liu Y. et al., 2020).

#### Social interaction

(Suspected) patients and survivors' social interaction [e.g., social activities (Liu Y. et al., 2020)] influences their stigma perceptions. One paper showed that only passive forms of social activities (e.g., having visitors at one's residence) contributed to perceived stigma toward suspected patients, while more proactive forms of social interaction did not [e.g., going to the grocery store or the pharmacy, and going to a friend's residence (Liu Y. et al., 2020)].

### Stigmatizer

Stigmatizers' characteristics affect their stigmatizing attitudes and behavior. We organize related studies into five sections: demographics, cognition, emotion, health, and social interaction (with the stigmatized group).

#### Demographics

Stigmatizers' demographics have been investigated in the literature (Abuhammad et al., 2021; Cassiani-Miranda et al., 2021; Hossain et al., 2021; Jiang et al., 2021; Li et al., 2021; Singh et al., 2021; Zhang et al., 2021). We identified two aspects highlighting the influence of stigmatizers' demographics: (1) certain demographics of stigmatizers are associated with less stigmatizing attitudes toward (suspected) patients and survivors because they reflect higher knowledge about COVID-19 (e.g., higher education and healthcare occupations vs. general public), and (2) certain demographics of stigmatizers are associated with more stigmatizing attitudes toward (suspected) patients and survivors because they reflect lower resources (e.g., rural vs. urban residence) and higher risk of COVID-19 infection (e.g., regions close to the origin location of COVID-19 and old age).

First, certain demographics of stigmatizers are associated with less stigmatizing attitudes toward (suspected) patients and survivors because they reflect higher knowledge about COVID-19 (e.g., higher education). For example, one study (Cassiani-Miranda et al., 2021) found that HCWs generally hold fewer stigmatizing attitudes toward COVID-19 patients compared with the general population. Another study (Hossain et al., 2021) found that individuals in Bangladesh with lower education backgrounds have higher levels of stigmatizing attitudes toward COVID-19 patients. Studies in Jordan (Abuhammad et al., 2021) and China (Zhang et al., 2021) also support this conclusion.

Second, certain demographics of stigmatizers are associated with more stigmatizing attitudes because they indicate lower resources to cope with COVID-19 (e.g., rural vs. urban residence) and higher COVID-19 risk (e.g., regions close to the origin location of COVID-19). For example, one study (Hossain et al., 2021) found that participants living in the rural and urban [excluding city corporation, i.e., “highly urbanized areas mostly in divisional headquarters” (Hossain et al., 2021, p. 3)] areas of Bangladesh tend to report higher stigmatizing attitudes toward people who tested positive for COVID-19 compared with participants living in city corporation areas. One study (Li et al., 2021) found that living in central China, which is close to the early epicenter of the pandemic, is associated with higher levels of discrimination against COVID-19 survivors. However, the results of age are rather mixed. While age has generally been found to be positively associated with stigma attitudes in studies conducted in China (Jiang et al., 2021; Li et al., 2021; Zhang et al., 2021), a study in Jordan (Abuhammad et al., 2021) found opposite results.

#### Cognition

Both stigmatizers' general cognition [e.g., feeling of resource scarcity, and trust in public officials (Chen et al., 2021a)] and COVID-19-related cognition [e.g., perceived risk/harm of COVID-19 (Hossain et al., 2021; Li et al., 2021), and vaccine attitudes (Li et al., 2021)] affect their stigmatizing attitudes toward (suspected) patients and survivors. Stigmatizers' general cognition influences their stigmatizing attitudes because stigmatizers who perceive low sense of control (e.g., feeling of resource scarcity, and low trust in public officials) tend to stigmatize (suspected) patients and survivors to restore their sense of control. COVID-19-related cognition influences stigmatizers' attitudes in two ways: (1) correct knowledge and perceptions about COVID-19 mitigate stigmatizing attitudes, and (2) stigmatizers who attribute more blame to (suspected) patients and survivors are more likely to stigmatize them.

For general cognition, stigmatizers whose cognition reflects a low sense of control (e.g., feeling of resource scarcity, and low trust in public officials) are more likely to stigmatize (suspected) patients and survivors as a way to restore their sense of control. For example, one study found that the feeling of resource scarcity is positively related to stigmatizing attitudes toward suspected patients [e.g., people from major outbreak sites, and people discharged from quarantine sites (Chen et al., 2021a)]. In addition, trust in public officials, which can be seen as a sense of security and control, is negatively associated with stigmatizing attitudes toward suspected patients (Chen et al., 2021a).

For COVID-19-related cognition, first, stigmatizers who have high levels of knowledge and correct perceptions about COVID-19 are less likely to stigmatize (suspected) patients and survivors. For example, one study found that Chinese people with a higher knowledge score about COVID-19 are less likely to hold stigmatizing attitudes toward COVID-19 survivors (Li et al., 2021). Misbeliefs and misconceptions about COVID-19 are generally found to contribute to stigmatizing attitudes (Ransing et al., 2020; Hossain et al., 2021; Zhang et al., 2021). One study found that the perceived magnitude of risk of COVID-19 is positively related to stigmatizing attitudes toward COVID-19 patients (Hossain et al., 2021). A study on vaccination and stigmatization found that both vaccine effectiveness and vaccination intention reduce stigmatizing attitudes (Li et al., 2021).

Second, stigmatizers who attribute more blame to (suspected) patients and survivors are more likely to stigmatize them. For example, one study found that Chinese people who attribute more personal responsibility to COVID-19 patients are more likely to adopt stigmatizing attitudes toward them (Zhang et al., 2021). In addition, a qualitative study suggests that blaming others for contracting the COVID-19 virus contributes to stigmatizing attitudes and behavior toward patients (Ransing et al., 2020).

#### Emotion

Stigmatizers' COVID-19-related emotions [e.g., fear (Ransing et al., 2020; Sorokin et al., 2020; Cassiani-Miranda et al., 2021; Chen et al., 2021a; Gopichandran and Subramaniam, 2021; Haddad et al., 2021; Zhang et al., 2021), and anxiety (Haddad et al., 2021)] influence their stigmatizing attitudes because stigmatization is used as a coping mechanism for negative emotions arising from the pandemic.

COVID-19 induces a lot of negative emotions (e.g., fear, anxiety, and anger), and individuals use stigmatization as a coping strategy to deal with these negative emotions (Chen et al., 2021b). For example, research has generally found that fear is positively associated with stigmatizing attitudes toward (suspected) patients and survivors (Sorokin et al., 2020; Cassiani-Miranda et al., 2021; Chen et al., 2021a; Gopichandran and Subramaniam, 2021; Haddad et al., 2021; Zhang et al., 2021). One study also found that feeling dangerous and angry toward COVID-19 patients is positively related to stigmatizing attitudes toward them (Zhang et al., 2021). However, another study found that other emotions (e.g., worry and optimism) are not related to stigmatization against suspected patients (Chen et al., 2021a).

#### Health

Stigmatizers' health may influence stigma because people in poor health may be more prone to be affected with COVID-19 (Chen N. et al., 2020) and may be more likely to stigmatize (suspected) patients and survivors. However, empirical studies did not find a significant relationship between stigmatizers' health and stigma. Specifically, one study found that self-rated health is not related to discrimination (Li et al., 2021). Another study also found that stigmatizers' own COVID-19 status is not related to stigmatizing attitudes toward COVID-19 patients (Hossain et al., 2021).

#### Social interaction (with the stigmatized group)

Stigmatizers' social interaction with (suspected) patients and survivors [e.g., infections of relatives and friends (Li et al., 2021), and direct or indirect contact with a (suspected) COVID-19 patient/survivor (Haddad et al., 2021)] influence their stigmatizing attitudes in two ways: (1) for stigmatizers who must interact with (suspected) patients and survivors (e.g., frontline workers), social interaction with the stigmatized group (e.g., serving patients) reduces their stigmatizing attitudes because they change their attitudes to resolve cognitive dissonance, and (2) social interaction with (suspected) patients and survivors reduces stigmatizing attitudes because stigmatizers gain more knowledge about COVID-19 and perceive less risk of the disease by interacting with the stigmatized group.

First, stigmatizers who must interact with (suspected) patients and survivors (e.g., frontline workers) may change their stigmatizing attitudes to resolve cognitive dissonance (Chen et al., 2021a). For example, one study found that the epidemic proximity of one's role, ranging from peripheral (e.g., general public) to intermediate (e.g., family members of COVID-19 patients) to center (e.g., HCWs, police), negatively predicts stigmatization and discrimination toward suspected patients (Chen et al., 2021a). A web-based cross-sectional study among HCWs also found that having served COVID-19 patients positively predicts acceptance of COVID-19 patients and negatively predicts fear of COVID-19 patients—two dimensions of stigma toward COVID-19 patients (Singh et al., 2021).

Second, having contact with (suspected) patients and survivors can increase stigmatizers' knowledge and correct perceptions of COVD-19, leading to decreased stigmatizing attitudes. For example, one study found that a history of COVID-19 in one's family and direct contact with a (suspected) COVID-19 patient/survivor are negatively related to stigmatizing attitudes toward patients (Haddad et al., 2021). However, one study found that knowing COVID-19 patients within their immediate social environment is not related to stigmatizing attitudes toward COVID-19 patients (Hossain et al., 2021). Another study also found that relatives' and friends' infections are not related to discrimination against COVID-19 survivors (Li et al., 2021).

### Context

Previous literature has investigated the influence of various contextual factors on stigma toward (suspected) patients and survivors. We organize these studies into three sections: media, policy, and COVID-19 situation.

#### Media

Media factors [e.g., (social) media use (Liu Y. et al., 2020; Yu et al., 2020), lack of public health information (Nursalam et al., 2020), and fake news (Sahoo and Patel, 2021)] can influence stigma toward (suspected) patients and survivors in two ways: (1) low-quality and poorly regulated messages in the media (e.g., misinformation, and irresponsible reporting) may create misconceptions about COVID-19, aggravating stigma toward (suspected) patients and survivors, and (2) (suspected) patients and survivors' frequent use of media may increase their stigma perceptions due to exposure to misinformation and stigmatizing messages in the media.

First, low-quality and poorly regulated messages (e.g., misinformation, and irresponsible reporting) contribute to stigma toward (suspected) patients and survivors by creating misconceptions about COVID-19. One qualitative study (Gopichandran and Subramaniam, 2021) and one review (Nursalam et al., 2020) showed that lack of COVID-19 information in the media creates fear and uncertainty, which aggravate stigma toward (suspected) patients and survivors. Misinformation and fake news in the media also create misconceptions about COVID-19, which also contribute to stigma toward (suspected) patients and survivors (Ransing et al., 2020; Gopichandran and Subramaniam, 2021; Sahoo and Patel, 2021). In addition, a qualitative study showed that patients tend to blame irresponsible media reporting (e.g., disclosure of personal information, and false allegation) as the main driver of stigma toward them (Jayakody et al., 2021).

Second, (suspected) patients and survivors' frequent social media use may be associated with increased perceived stigma due to increased exposure to misinformation and stigmatizing messages in the media. One study found that residents who spend more than 2 h per day on average on social media report higher levels of stigma experiences because of their suspected COVID-19 status compared with residents who use social media less often (Liu Y. et al., 2020). However, another study did not find a significant relationship between social media use and perceived stigma from COVID-19 (Yuan et al., 2021).

#### Policy

Policy-related factors [e.g., involvement of police (Gopichandran and Subramaniam, 2021), different and conflicting information about COVID-19 and quarantine procedures (Lohiniva et al., 2021)] influence stigma toward (suspected) patients and survivors. The influence of policy-related factors can be understood in two ways: (1) conflicting and inconsistent COVID-19 policies may create uncertainty for (suspected) patients and survivors of their health risks, resulting in higher self-stigma, and (2) inappropriate implementation of health policies may aggravate negative perceptions of COVID-19, which leads (suspect) patients to perceive more stigma.

First, conflicting and inconsistent COVID-19 policies increase (suspected) patients' and survivors' uncertainty about their health risks, leading to self-stigma (Lohiniva et al., 2021). For example, one study found that different and conflicting information about quarantine procedures positively predicts (suspected) patients' self-stigma during quarantine and isolation (Lohiniva et al., 2021).

Second, inappropriate implementation of health policies may aggravate negative perceptions of (suspected) patients and survivors, resulting in more stigma perceptions in these individuals. For example, a qualitative study showed that police involvement in the enforcement of COVID-19 health policies “made people associate COVID-19 with crime” (Gopichandran and Subramaniam, 2021, p. 196), leading to more stigma perceptions. In addition, forced isolation policies also contribute to stigma perceptions among (suspected) patients and survivors (Gopichandran and Subramaniam, 2021).

#### COVID-19 situation

The COVID-19 situation (i.e., number of confirmed COVID-19 cases in a province) influences stigmatizing attitudes toward (suspected) patients and survivors. One study found that living in a province with more confirmed cases is associated with more stigma toward (suspected) COVID-19 patients and survivors probably because having more confirmed cases heightens infection risk, especially in China, where most provinces are categorized as low to medium risk (Jiang et al., 2021).

## Antecedents of stigma toward HCWs

### The stigmatized

Based on literature review, we organize the influences of the stigmatized on their perceived stigma into four sections: demographics, work, health, and coping.

#### Demographics

HCWs' demographics (Yadav et al., 2020; Zandifar et al., 2020; Adhikari et al., 2021; Radhakrishnan et al., 2021; Uvais et al., 2021; Khan et al., 2022) have been investigated in the literature. The main logic is that HCWs' demographics that reflect higher health risks (e.g., staying at the hospital) are associated with increased perceived stigma. For example, one study (Yadav et al., 2020) found that HCWs who stay in a hostel or accommodations provided by a hospital report higher perceived stigma than those who stay at home. Another study (Adhikari et al., 2021) found that HCWs living with the elderly, who are more prone to infection (Mueller et al., 2020), report higher perceived stigma. However, two studies found no difference in perceived stigma between living with family or not (Radhakrishnan et al., 2021; Uvais et al., 2021).

It is noteworthy that though some scholars suggest that female HCWs may be more vulnerable to stressful environments and more prone to feeling stigmatized (Zandifar et al., 2020), other studies found a null association between gender and perceived stigma (Yadav et al., 2020; Adhikari et al., 2021; Radhakrishnan et al., 2021; Uvais et al., 2021; Khan et al., 2022).

#### Work

HCWs' work characteristics [e.g., frontline worker (Adhikari et al., 2021; Khan et al., 2022), and work area (Radhakrishnan et al., 2021)] have been investigated in the literature. HCWs' work characteristics that indicate high risk of infection (e.g., frontline HCWs, and clinical vs. non-clinical areas) are associated with higher perceived stigma. For example, two studies found that HCWs working on the front lines report higher perceived stigma (Zandifar et al., 2020; Adhikari et al., 2021). Another study found that HCWs who work in clinical areas (vs. non-clinical areas) report more perceived stigma (Radhakrishnan et al., 2021). Designations reflecting potential contact with the COVID-19 virus, such as resident doctors (vs. faculty/medical officers, Yadav et al., 2020) and surgical department (vs. medicine and critical care, Yadav et al., 2020), have been found to be associated with higher levels of perceived stigma. However, one study found that HCWs working in high-risk vs. low-risk areas do not differ in perceived stigma (Yadav et al., 2020). Moreover, the findings on occupations are rather mixed. For example, one study found that compared with doctors, nurses are more likely to experience stigma (Radhakrishnan et al., 2021), while another found opposite results (Zandifar et al., 2020).

#### Health

HCWs' health conditions [e.g., being diagnosed with COVID-19 (Adhikari et al., 2021)] can influence perceived stigma because poor health may increase HCWs' fear of infection and anticipated stigma. One study found that only being diagnosed with COVID-19, rather than other health conditions (e.g., being admitted to the hospital due to COVID-19, and chronic diseases), is positively related to perceived stigma among HCWs (Adhikari et al., 2021).

#### Coping

HCWs' use of coping strategies [e.g., precautionary measures (Adhikari et al., 2021), and quarantine (Uvais et al., 2021; Khan et al., 2022)] influence perceived stigma for two reasons: (1) HCWs who take sufficient precautionary measures may feel safer and less concerned about potential stigma from patients, leading to decreased perceived stigma, and (2) HCWs who are quarantined may perceive themselves as being at high risk of transmitting the disease, resulting in more perceived stigma.

First, sufficient precautionary measures may decrease HCWs' concerns about and fear of infection and stigma from patients, leading to decreased perceived stigma. For example, one study found that HCWs who believe the precautionary measures they are taking are sufficient (vs. insufficient) tend to perceive less stigma (Adhikari et al., 2021).

Second, quarantine signals health risks, which may make HCWs perceive more stigma toward themselves. For example, one study found that being in quarantine is positively related to perceived stigma among physicians in Bangladesh (Khan et al., 2022). However, another study found that a history of quarantine is not related to doctors' perceived stigma (Uvais et al., 2021).

### Stigmatizer

Scholars also investigated how stigmatizers' characteristics affect stigma toward HCWs, and we group these studies into three sections: demographics, cognition, and emotions.

#### Demographics

Stigmatizers' demographics have been examined in the literature (Cassiani-Miranda et al., 2021). Stigmatizers' demographics that indicate higher knowledge of COVID-19 (e.g., HCW occupation vs. general public) are associated with less stigmatizing attitudes. Indeed, one study found that compared with HCWs, individuals in the general population are more likely to adopt stigmatizing attitudes toward HCWs (Cassiani-Miranda et al., 2021).

#### Cognition

Stigmatizers' cognition, both general and COVID-19 related, affects stigmatizing attitudes and behavior. Specifically, general cognition that reflects a low sense of control (e.g., feeling of resource scarcity, and low trust in public officials, Chen et al., 2021a) is positively related to stigma toward HCWs as some individuals use stigmatization as a coping mechanism to restore their sense of control. COVID-19-related cognition that reflects dehumanization (e.g., objectification of people returning from Hubei, Chen et al., 2021a) is associated with indifference and instrumental orientation toward affected people, which contributes to stigma toward HCWs.

For general cognition, stigmatizers whose cognition reflects a low sense of control are more likely to stigmatize HCWs as a way to restore their sense of control. For example, one study found that the feeling of resource scarcity and the need to belong are positively related to stigmatization and discrimination toward HCWs (Chen et al., 2021a). At the same time, trust in health experts, but not trust in public officials or the general public, is negatively associated with stigmatization and discrimination toward HCWs.

For COVID-19-related cognition, stigmatizers' objectification cognition denies individuals' humanity and leads to perceptions of people as “possible sources of infection” (Chen et al., 2021a, p 8), which contributes to stigma toward HCWs. Indeed, one study found that the objectification of people returning to Hubei is a powerful predictor for stigmatization and discrimination toward HCWs (Chen et al., 2021a).

#### Emotion

Research on stigmatizers' emotions has focused on COVID-19-related emotions, such as fear (Taylor et al., 2020; Cassiani-Miranda et al., 2021; Chen et al., 2021a) and stress (Taylor et al., 2020) toward COVID-19. These emotions affect stigma because stigmatization is used as a coping mechanism for negative emotions arising from the pandemic. For example, research has found that fear (Taylor et al., 2020; Cassiani-Miranda et al., 2021; Chen et al., 2021a) and stress (Taylor et al., 2020) toward COVID-19 are positively associated with stigma toward HCWs. However, other studies did not find a significant relationship between other emotions (e.g., optimism, Chen et al., 2021a) and stigma^1^.

## Outcomes of stigma toward Chinese/Asian people

For each stigma target, we categorize the outcomes of COVID-19-related stigma into two broad sections: health and non-health outcomes.

### Health

We organize health outcomes of stigma toward Chinese/Asian people into mental and physical health.

#### Mental health

Empirical studies have investigated the influence of stigma on Chinese/Asian people's mental health, such as depression, anxiety, and mental disorders. Stigma negatively influences Chinese/Asian people's mental health because stigma communicates hostility and diminishes the value of stigmatized groups, which threatens their sense of identity, control, and self-worth. Indeed, findings generally show that stigma toward Chinese/Asian people is associated with depression (Cheah et al., 2020; Litam and Oh, 2020, 2021; Hahm et al., 2021a,b; Lee and Waters, 2021), anxiety (Cheah et al., 2020; Yang and Wong, 2020; Adhikari et al., 2021; Chen X. et al., 2021; Haft and Zhou, 2021; Hahm et al., 2021a,b; Lee and Waters, 2021), fear (Hahm et al., 2021b), mental disorders (Wu C. et al., 2021), psychological distress (Chen et al., 2021b; Fan et al., 2021; Maglalang et al., 2021), anger (Chen X. et al., 2021), grief (Chen X. et al., 2021), internalizing and externalizing problems (Cheah et al., 2020), post-traumatic stress disorder (PTSD) (Hahm et al., 2021a), and decreased psychological well-being (Cheah et al., 2020), life satisfaction (Litam and Oh, 2020, 2021), and self-esteem (Alsawalqa, 2021). For example, one study found that COVID-19-related racial discrimination, especially the in-person direct form, is negatively associated with multiple adverse mental health indices for both Chinese American parents and youth, such as depressive symptoms, anxiety symptoms, and poor psychological wellbeing (Cheah et al., 2020).

#### Physical health

Research found that stigma affects Chinese/Asian people's physical health (e.g., sleep difficulties and self-rated health) because poor mental health resulting from stigma perceptions may further negatively impact their physical health. Indeed, one study found that racial discrimination during the COVID-19 pandemic is positively related to sleep difficulties and physical symptoms (e.g., headaches) in a sample of Asians and Asian Americans (Lee and Waters, 2021). They further found that social support buffers against the negative impact of discrimination on physical symptoms but not on sleep difficulties.

### Non-health

The non-health outcomes of stigma toward Chinese/Asian people are mainly social activity-related outcomes.

#### Social activity

Studies have found that perceived stigma affects Chinese/Asian people's social activities and social life (e.g., avoidance of social activities). Two mechanisms explain the social impacts of perceived stigma on Chinese/Asian people: (1) Chinese/Asian people who perceive higher stigma tend to avoid in-person social interaction and prefer interacting in virtual spaces because their stigmatized identity is more salient offline, and (2) Chinese/Asian people who perceive stigma tend to engage in altruistic behavior to improve their group's image.

First, Chinese/Asian people who perceive higher stigma tend to avoid in-person social interaction but not online interaction because they fear disclosing their stigmatized identity in real life. For example, a qualitative study showed that one of the outcomes of COVID-19-related anti-Asian discrimination is avoidance (Hahm et al., 2021b). One study found that perceived discrimination since the COVID-19 outbreak is negatively related to interacting with friends, indicating withdrawal from social activities (Chen et al., 2021b). In addition, another study found that experiencing discrimination is positively related to social media use, including posting, commenting, browsing, and private messaging (Yang et al., 2020).

Second, Chinese/Asian people who perceive higher stigma tend to engage in altruistic behavior to improve their group's image. For example, one study found that perceived stigma is positively related to altruistic tendencies through emotional mechanism (e.g., grief) in a Chinese sample (Chen X. et al., 2021).

## Outcomes of stigma toward (suspected) patients and survivors

### Health

The health outcomes of stigma toward (suspected) patients and survivors can be categorized into mental and physical health.

#### Mental health

The influence of stigma on (suspected) patients' and survivors' mental health (e.g., depression, feelings of loneliness, and wellbeing) has received much research attention. Stigma negatively affects (suspected) patients' and survivors' mental health because the stigmatized perceive themselves as alienated, isolated, and belonging to a devalued group and perceive stigma as unfair. Indeed, empirical findings generally show that stigma harms (suspected) patients' and survivors' mental health and leads to issues like depression (Liu D. et al., 2020; Xin et al., 2020; Grover et al., 2021; Harjana et al., 2021; Jayakody et al., 2021; Kang et al., 2021; Yuan et al., 2021), anxiety (Liu D. et al., 2020; Atinga et al., 2021; Gopichandran and Subramaniam, 2021; Grover et al., 2021; Harjana et al., 2021; Jayakody et al., 2021; Kang et al., 2021), mental health disorders (Adom et al., 2021; Miconi et al., 2021a; Paleari et al., 2021), lower well-being (Ransing et al., 2020; Al Eid et al., 2021; Paleari et al., 2021; Sahoo and Patel, 2021), PTSD (Liu D. et al., 2020; Grover et al., 2021; Kang et al., 2021; Mahmoudi et al., 2021; Poyraz et al., 2021; Gan et al., 2022), and even self-harm or suicide (Ransing et al., 2020; Xin et al., 2020; Sahoo and Patel, 2021). For example, in a large sample involving more than 20,000 Chinese students, one study found that perceived discrimination due to COVID-19 is positively related to self-harm or suicidal ideation (Xin et al., 2020).

#### Physical health

Stigma toward (suspected) patients and survivors also harms their physical health (Earnshaw et al., 2020; Ransing et al., 2020; Atinga et al., 2021; Bhanot et al., 2021; Lohiniva et al., 2021; Mahmoudi et al., 2021; Poyraz et al., 2021). The influence of stigma toward (suspected) patients and survivors on their physical health can be understood in two ways: (1) (suspected) patients and survivors' poor mental health resulting from perceived stigma further deteriorates their physical health conditions, and (2) (suspected) patients and survivors try to avoid stigma by concealing COVID-19-related symptoms and delaying treatment, which threatens one's own physical health.

First, (suspected) patients and survivors' poor mental health resulting from perceived stigma negatively impacts their physical health conditions. For example, one study found that self-stigma is associated with insomnia and poor physical quality of life *via* poor mental health (Mahmoudi et al., 2021). A qualitative study also documented various adverse physical health consequences for patients who have experienced stigma, such as delays in recovery, insomnia, and eating disorders (Atinga et al., 2021).

Second, (suspected) patients and survivors try to avoid stigma by concealing COVID-19-related symptoms and delaying treatment, which threatens one's own physical health. For example, one study found that stigma toward COVID-19 patients—both anticipated stigma and stereotypes—is negatively related to COVID-19 testing because people want to avoid being labeled as having the stigmatized disease (Earnshaw et al., 2020). Another study showed that one of the outcomes of perceived stigma among patients is reluctance to disclose their COVID-19 status (Lohiniva et al., 2021). Moreover, delayed testing and concealment of symptoms could hinder progress toward controlling COVID-19 and thereby threaten public health (Bhanot et al., 2021; Lohiniva et al., 2021).

### Non-health

Research on the non-health outcomes of stigma toward (suspected) patients and survivors has focused on three aspects: social activity, work, and coping.

#### Social activity

Stigma toward (suspected) patients and survivors negatively affects their social interaction (Atinga et al., 2021; Lohiniva et al., 2021) because they worry about and fear being stigmatized when disclosing their COVID-19 status during interactions. For example, one study interviewed COVID-19 patients and found that social withdrawal is a common consequence of experiencing stigma (Atinga et al., 2021).

#### Work

Stigma toward (suspected) patients and survivors may negatively affect their work outcomes *via* lowering work motivation and engagement (Ransing et al., 2020). Stigma toward (suspected) patients and survivors may aggravate poverty and social inequality because the stigmatized may suffer from unemployment (Ransing et al., 2020).

#### Coping

Stigma may have two-sided effects on (suspected) patients' and survivors' coping strategies: (1) (suspected) patients and survivors increase their compliance with health guidelines because they hope to eliminate the risk of transmitting COVID-19 and end their stigmatization; (2) they may reduce their use of coping strategies to avoid being identified and stigmatized. For example, a qualitative study found that increased compliance with health guidelines is a strategy used by (suspected) patients to avoid stigma (Lohiniva et al., 2021). However, a review paper discussed that due to fear of being stigmatized, (suspected) patients may avoid using coping strategies, such as self-isolation (Saeed et al., 2020).

## Outcomes of stigma toward HCWs

### Health

Studies on the health outcomes of stigma toward HCWs have examined both mental and physical health outcomes.

#### Mental health

Empirical findings generally show that stigma toward HCWs harms their mental health [e.g., depression (Elhadi et al., 2020; Khanal et al., 2020; Teksin et al., 2020; Campo-Arias et al., 2021; Greene et al., 2021; Kirk et al., 2021), anxiety (Elhadi et al., 2020; Khanal et al., 2020; Teksin et al., 2020; Campo-Arias et al., 2021; Greene et al., 2021; Kirk et al., 2021), burnout (Patel et al., 2021; Shiu et al., 2022), and suicide risk (Campo-Arias et al., 2021)]. The negative impacts of stigma on HCWs may be due to compromised work meaningfulness and diminished self-esteem and self-worth. For example, one study found that perceived stigma is significantly associated with anxiety, depression, and PTSD (Teksin et al., 2020). Two other studies also showed that perceived stigma is positively related to burnout among HCWs (Patel et al., 2021; Shiu et al., 2022).

#### Physical health

The negative influence of stigma on HCWs' mental health may further affect their physical health (Khanal et al., 2020; Teksin et al., 2020). For example, one study found that experienced stigma is positively related to insomnia among HCWs (Khanal et al., 2020).

### Non-health

Studies on the non-health outcomes of stigma toward HCWs can be organized into two sections: work and coping outcomes.

#### Work

Stigma toward HCWs may increase their turnover intention as a way to avoid the stigmatized identity. For example, in a sample of frontline nurses, one study found that perceived COVID-19-associated discrimination is positively related to turnover intention (Labrague et al., 2021).

#### Coping

Stigma affects HCWs' coping strategies because they may increase the use of maladaptive coping strategies to manage the negative influence of stigma. A qualitative study found that increased alcohol use was one response to being stigmatized (Zolnikov and Furio, 2020).

## Conclusion

Based on our review, we present three major findings in this paper. First, we identify the three most researched targets of COVID-19-related stigma: Chinese/Asian people, (suspected) patients and survivors, and healthcare workers (HCWs). Second, we reveal that for each stigma target, research on the antecedents of COVID-19-related stigma has examined characteristics of the stigmatized, stigmatizer, and context. We organize the characteristics of the stigmatized into seven sections (i.e., demographics, work, cognition, emotion, health, coping, and social interaction), the characteristics of stigmatizer into six sections (i.e., demographics, cognition, emotion, health, coping, and social interaction), and the characteristics of context into four sections (i.e., culture, media, policy, and COVID-19 situation). Third, we find that COVID-19-related stigma has severe negative impacts on victims' health (i.e., mental and physical health outcomes) and non-health outcomes (i.e., social activity, work, and coping).

## Suggestions for research

This review aims to assess the antecedents and consequences of COVID-19-related stigma toward three groups: Chinese/Asian people, (suspected) patients and survivors, and HCWs. We identified three areas for improvement and call on future research to expand the current focus and use rigorous research methods to examine the rather neglected research topics associated with COVID-19-related stigma.

First, future research should provide a more balanced picture of the targets, antecedents, and consequences of COVID-19-related stigma. From our review, the distribution of research on these topics is uneven. Specifically, for the stigmatized targets, current research has paid more attention to (suspected) patients and survivors as the victims of COVID-19-related stigma compared to the other two targets (i.e., Chinese/Asian people and HCWs), which may limit our comprehensive understanding of COVID-19-related stigma. Thus, we urge more future work to examine Chinese/Asian people and HCWs as the stigmatized targets. In addition, HCWs constitute a unique target of COVID-19-related stigma because they are being stigmatized as well as being recognized at the same time (Nashwan et al., 2022a), which may require further theorizing and investigation about their stigma-related psychological process. For the antecedents, research targeting Chinese/Asian people has evenly focused on exploring the antecedents of the stigmatized, stigmatizer, and context, while research targeting (suspected) patients and survivors has focused more on antecedents of stigmatizer and research targeting HCWs has tended to ignore the antecedents of context, which may limit our complete understanding of how to reduce or even prevent COVID-19-related stigma. Indeed, a large-scale study of 1,726 health care providers across seven countries shows that their nationality is significantly associated with all items of the Stigma COVID-19 Healthcare Providers tool, which suggests that national level differences may contribute to HCWs' COVID-19-related stigma perception (Nashwan et al., 2022b). Thus, we urge more future work to explore the lesser noticed antecedents—for example, third-party's reactions to discriminatory and prejudicial behaviors posed by stigmatizers may serve as a helping hand for victims and potentially reduce COVID-19-related stigma (Liu et al., 2021). For the outcomes, research has mainly investigated the health outcomes (especially mental health) of COVID-19-related stigma at the expense of ignoring important non-health outcomes, which may overlook the opportunity to detect social problems that result from COVID-19-related stigma at early stage. Thus, we urge future work to explore the non-health effects of COVID-19-related stigma for all three groups. For example, future research may examine how COVID-19-related stigma affects these groups' economic, work, and family outcomes.

Second, future research should investigate interventions for COVID-19-related stigma. Researchers have generally acknowledged that interventions are a vital and effective way to decrease COVID-19-related stigma in the discussion sections of their articles. However, there has been little empirical evidence evaluating the effectiveness of such interventions (Teksin et al., 2020; Gronholm et al., 2021; Valeri et al., 2021), which may make it difficult to provide evidence-based practice or policy suggestions for how to reduce COVID-19-related stigma. As an exceptional example, one study showed that HCWs' emotion-focused and problem-focused coping strategies are negatively related to stigma perceptions (Teksin et al., 2020). Thus, we suggest that future research conduct field experiments to propose interventions on COVID-19-related stigma and evaluate the effects of these interventions. For example, future research could draw on our review and explore how interventions aimed at different aspects of antecedents could help mitigate COVID-19-related stigma (e.g., for the stigmatized: psychological counseling or training; for stigmatizer: interaction with the stigmatized; for context: legislation). In addition, future research may draw on the PICO (patient/population, intervention, comparison and outcomes) framework to examine if one intervention is superior to another intervention in reducing COVID-19-related stigma for a specific target.

Third, future research could conduct more longitudinal studies of COVID-19-related stigma. By reviewing the extant literature, we found that most research has used cross-sectional or qualitative data, while few studies have used longitudinal data to examine the long-term effects of COVID-19-related stigma, which limits our understanding of the causal relationships between COVID-19-related antecedents and stigma and between COVID-19-related stigma and outcomes. In addition, the use of cross-sectional data fails to capture the fluctuations and trends of COVID-19-related stigma. An exceptional example showed that perceived discrimination due to COVID-19 increased from mid-March to mid-April 2020 and then decreased significantly by early June 2020 (Robinson and Daly, 2021). Thus, longitudinal studies are needed to examine the trajectory of COVID-19-related stigma, which may inform research and practice on what contributes to COVID-19-related stigma changes and its consequences.

## Suggestions for institutions (especially governments)

Our review also offers three suggestions for governments and institutions on how to deal with COVID-19-related stigma. First, governments and institutions should pay close attention to the stigma issue during the pandemic. Our review showed that COVID-19 not only is a major public health issue but also engenders serious secondary threats to broader society (Yam et al., 2020; Harjana et al., 2021; Lee and Waters, 2021) because the stigma that accompanies COVID-19 also brings serious health and non-health consequences for victims spanning multiple groups and countries (Duan et al., 2020; Xu et al., 2021). Therefore, we call for governments and institutions to pay close attention not only to the treatment and prevention of COVID-19 as a disease but also to the problem of and solutions for COVID-19-related stigma. For example, the ministries of public health could focus more on disseminating reliable and updated health information to the public to restore people's confidence in interacting with other people and alleviate COVID-19-related stigma. In addition, hospitals and communities could provide follow-up consultations for COVID-19 survivors after their discharge.

Second, governments and institutions should protect the personal information of stigmatized groups. Our findings revealed that once individuals are stigmatized, their health and lives suffer greatly. This problem may be especially severe for certain groups that lack resources and are more vulnerable to COVID-19-related stigma, such as people with low SES, and minorities. As a result, COVID-19-related stigma could result in a widened gap between the rich and the poor as well as aggravated social inequality. Therefore, we believe that the first step governments and institutions could take is to protect stigmatized targets' personal information and avoid disclosing any personally identifiable information that would lead to these people being identified and stigmatized. For example, the ministries of justice and the ministries of home affairs could implement laws, public policies, or guidance on restrictions of collecting and using personal information to protect targets' privacy. Similarly, officially published COVID-19 information should be de-identified and anonymized.

Third, we urge both traditional and social media outlets to take responsibility for alleviating stigma toward all groups. Our findings revealed that due to the prevalence of low-quality content in media, usage of media is generally found to be positively related to stigma for both stigmatizer and the stigmatized (Croucher et al., 2020; Dhanani and Franz, 2020, 2021; Duan et al., 2020; Tsai et al., 2020; Yu et al., 2020; Cho et al., 2021; Haft and Zhou, 2021). Therefore, we urge both traditional and social media outlets to take responsibility for their journalistic professionalism, ensuring that they publish correct, fair, and scientific information and avoid using stigmatizing words. In addition, the algorithms used by social media outlets to create newsfeeds may contribute to stigma by reinforcing biased beliefs or negative judgments toward stigmatized groups (Dhanani and Franz, 2020; Duan et al., 2020; Cho et al., 2021). Therefore, we propose that social media platforms improve current algorithms that may exacerbate the problem of opinion polarization and even work on developing algorithms and new technologies that can control the spread of fake news and stigmatizing messages on social media.

## Limitations

Our results may be subject to limitations related to the selection process of eligible studies. We did not assess the quality of papers that are included in our literature review, which may limit the validity of our results. However, including studies in a variety of sources could reduce publication bias (Song et al., 2013) and provide a broader and more representative overview of the COVID-19-related stigma issue. Therefore, studies that are published in outlets other than the first-tier journals are also included in our systematic review. In addition, our search is based on Google Scholar. Although Google Scholar offers a wide range of sources and is widely used in other review work (Haddaway et al., 2015), we may overlook eligible studies by solely using it as our database. Furthermore, we exclude studies that are in languages other than English, which could lead to a potential risk of bias. Coupled with studies published in other languages and databases, we hope future research to move closer to a more well-rounded understanding of the COVID-19-related stigma issue.

## Data availability statement

The original contributions presented in the study are included in the article/Supplementary material, further inquiries can be directed to the corresponding author.

## Author contributions

XZ, CC, YY, JX, and XQ contributed to the conception, design, and implementation of this research. XZ, CC, YY, JX, LC, and XQ contributed to the writing of the manuscript. All authors contributed to the article and approved the submitted version.

## Funding

This research was supported by two grants funded by National Natural Science Foundation of China (Grant Nos. 72272155 and 71872190), awarded to XQ, a grant funded by National Natural Science Foundation of China (Grant No. 71702202), and Fundamental Research Funds for the Central Universities (Grant No. 19wkpy17), awarded to CC.

## Conflict of interest

The authors declare that the research was conducted in the absence of any commercial or financial relationships that could be construed as a potential conflict of interest.

## Publisher's note

All claims expressed in this article are solely those of the authors and do not necessarily represent those of their affiliated organizations, or those of the publisher, the editors and the reviewers. Any product that may be evaluated in this article, or claim that may be made by its manufacturer, is not guaranteed or endorsed by the publisher.

## Footnotes

^1^We did not include context section for HCWs due to lack of research.
