# Supplementary material for: The scar that takes time to heal: A systematic review of COVID-19-related stigma targets, antecedents, and outcomes
Source: Front Psychol. 2022 Dec 1;13:1026712. doi: 10.3389/fpsyg.2022.1026712 (PMC9752089; doi:10.3389/fpsyg.2022.1026712)
Supplement: Supplementary file 1 [file Presentation_1.pdf]

Appendix

Studies Included in the Systematic Review

Abuhammad, S., Alzoubi, K. H., and Khabour, O. (2021). Fear of COVID-19 and stigmatization towards infected people among Jordanian people. *Int. J. Clin. Pract.* 75, e13899. doi: 10.1111/ijcp.13899

Adhikari, S. P., Rawal, N., Shrestha, D. B., Budhathoki, P., Banmala, S., Awal, S., et al. (2021). Prevalence of anxiety, depression, and perceived stigma in healthcare workers in nepal during later phase of first wave of covid-19 pandemic: A web-based cross-sectional survey. *Cureus* 13, e16037. doi: 10.7759/cureus.16037

Adom, D., Mensah, J. A., and Osei, M. (2021). The psychological distress and mental health disorders from COVID-19 stigmatization in Ghana. *Soc. Sci. Humanit. Open* 4, 100186. doi: 10.1016/j.ssaho.2021.100186

Ahmed, S., Chen, V. H. H., and Chib, A. I. (2021). Xenophobia in the time of a pandemic: Social media use, stereotypes, and prejudice against immigrants during the COVID-19 crisis. *Int. J. Public Opin. Res.* 33, 637-653. doi: 10.1093/ijpor/edab014

Al Eid, N. A., Arnout, B. A., Alqahtani, M. M., and Fadhel, F. H. (2021). The mediating role of religiosity and hope for the effect of self-stigma on psychological well-being among COVID-19 patients. *Work* 68, 525-541. doi: 10.3233/WOR-203392

Alatrany, S. S. J. (2020). COVID-19 related stigma, examining the view of the general public of stigma toward people with COVID-19 in Iraq. *Int. J. Psychosoc. Rehabil.* 24, 7108-7115.doi:10.37200/IJPR/V24I5/PR2020720

Alsawalqa R. O. (2021). Cyberbullying, social stigma, and self-esteem: The impact of COVID-19 on students from East and Southeast Asia at the University of Jordan. *Heliyon* 7, e06711. doi: 10.1016/j.heliyon.2021.e06711

Al-Zamel, L. A., Al-Thunayan, S. F., Al-Rasheed, A. A., Alkathiri, M. A., Alamri, F., Alqahtani, F., et al. (2021). Validation and cultural adaptation of explanatory model interview catalogue (EMIC) in assessing stigma among recovered patients with COVID-19 in Saudi Arabia. *Int. J. Environ. Res. Public Health* 18, 8261. doi: 10.3390/ijerph18168261

Atinga, R. A., Alhassan, N. M. I., and Ayawine, A. (2021). Recovered but constrained: Narratives of Ghanaian COVID-19 survivors experiences and coping pathways of stigma, discrimination, social exclusion and their sequels. *Int. J. Health Policy Manage.* 11, 1801-1813. doi: 10.34172/ijhpm.2021.81

Bhanot, D., Singh, T., Verma, S. K., and Sharad, S. (2021). Stigma and discrimination during COVID-19 pandemic. *Front. Public Health* 8, 577018. doi: 10.3389/fpubh.2020.577018

Campo-Arias, A., Jiménez-Villamizar, M. P., and Caballero-Domínguez, C. C. (2021). Healthcare workers' distress and perceived discrimination related to COVID-19 in Colombia. *Nurs. Health Sci.* 23, 763-767. doi: 10.1111/nhs.12854

Cassiani-Miranda, C. A., Campo-Arias, A., Tirado-Otalvaro, A. F., Botero-Tobon, L. A., Upegui-Arango, L. D., Rodriguez-Verdugo, M. S., et al. (2021). Stigmatisation associated with COVID-19 in the general Colombian population. *Int. J. Soc. Psychiatr.* 67, 728-736. doi: 10.1177/0020764020972445

Cheah, C. S., Wang, C., Ren, H., Zong, X., Cho, H. S., and Xue, X. (2020). COVID-19 racism and mental health in Chinese American families. *Pediatr.* 146, e2020021816. doi: 10.1542/peds.2020-021816

Cheah, C. S. L., Zong, X., Cho, H. S., Ren, H., Wang, S., Xue, X., and Wang, C. (2021). Chinese American adolescents' experiences of COVID-19 racial discrimination: Risk and protective factors for internalizing difficulties. *Cultural Diversity Ethn. Minority Psychol.* 27, 559–568. doi: 10.1037/cdp0000498

Chen, X., Gao, H., Zou, Y., and Lin, F. (2020). Changes in psychological wellbeing, attitude and information-seeking behaviour among people at the epicentre of the COVID-19 pandemic: A panel survey of residents in Hubei province. *China. Epidemiol. Infect.* 148, e201. doi: 10.1017/S0950268820002009

Chen, X., Huang, C., Wang, H., Wang, W., Ni, X., and Li, Y. (2021). Negative emotion arousal and altruism promoting of online public stigmatization on COVID-19 pandemic. *Front. Psychol.* 12, 652140. doi: 10.3389/fpsyg.2021.652140

Chen, Y., Jin, J., Zhang, X., Zhang, Q., Dong, W., and Chen, C. (2021). Reducing objectification could tackle stigma in the COVID-19 pandemic: Evidence from China. *Front. Psychol.* 12, 664422. doi: 10.3389/fpsyg.2021.664422

Chen, Y., Wang, Z., Dong, W., Xu, J. H. C., Wu, S. J., Zhang, X., et al. (2021). The pathways from perceived discrimination to self-rated health among the Chinese diaspora during the COVID-19 pandemic: Investigation of the roles of depression, anxiety, and social support. *Int. J. Equity Health* 20, 192. doi: 1186/s12939-021-01537-9

Cho, H., Li, W., Cannon, J., Lopez, R., and Song, C. (2021). Testing three explanations for stigmatization of people of Asian descent during COVID-19: Maladaptive coping, biased media use, or racial prejudice? *Ethn. Health* 26, 94-109. doi: 10.1080/13557858.2020.1830035

Croucher, S. M., Nguyen, T., and Rahmani, D. (2020). Prejudice toward Asian Americans in the COVID-19 pandemic: The effects of social media use in the United States. *Front. Commun.* 5, 39. doi: 10.3389/fcomm.2020.00039

Dar, S. A., Khurshid, S. Q., Wani, Z. A., Khanam, A., Haq, I., Shah, N. N., et al. (2020). Stigma in coronavirus disease-19 survivors in Kashmir, India: A cross-sectional exploratory study. *PLoS ONE* 15, e0240152. doi: 10.1371/journal.pone.0240152

Dhanani, L. Y., and Franz, B. (2020). The role of news consumption and trust in public health leadership in shaping COVID-19 knowledge and prejudice. *Front. Psychol.* 11, 560828. doi: 10.3389/fpsyg.2020.560828

Dhanani, L. Y., and Franz, B. (2021). Why public health framing matters: An experimental study of the effects of COVID-19 framing on prejudice and xenophobia in the United States. *Soc. Sci. Med.* 269, 113572. doi: 10.1016/j.socscimed.2020.113572

Duan, W., Bu, H., and Chen, Z. (2020). COVID-19-related stigma profiles and risk factors among people who are at high risk of contagion. *Soc. Sci. Med.* 266, 113425. doi: 10.1016/j.socscimed.2020.113425

Earnshaw, V. A., Brousseau, N. M., Hill, E. C., Kalichman, S. C., Eaton, L. A., and Fox, A. B. (2020). Anticipated stigma, stereotypes, and COVID-19 testing. *Stigma Health* 5, 390–393. doi: 10.1037/sah0000255

Elhadi, M., Msherghi, A., Elgzairi, M., Alhashimi, A., Bouhuwaish, A., Biala, M., et al. (2020). Psychological status of healthcare workers during the civil war and COVID-19 pandemic: A cross-sectional study. *J. Psychosom. Res.* 137, 110221. doi: 10.1016/j.jpsychores.2020.110221

Fan, W., Qian, Y., and Jin, Y. (2021). Stigma, perceived discrimination, and mental health during China's COVID-19 outbreak: A mixed-methods investigation. *J. Health Soc. Behav.* 62, 562–581. doi.org/10.1177/00221465211040550

Gan, Y., Ma, J., Wu, J., Chen, Y., Zhu, H., and Hall, B. J. (2022). Immediate and delayed psychological effects of province-wide lockdown and personal quarantine during the COVID-19 outbreak in China. *Psychol. Med.* 52, 1321–1332. doi: 10.1017/S0033291720003116

Gardner, D. M., Briggs, C. Q., and Ryan, A. M. (2021). It is your fault: workplace consequences of anti-Asian stigma during COVID-19. *Equality Diversity Inclusion: Int. J.* 41, 3-18.doi: 10.1108/EDI-08-2020-0252

Gazi, A. K. Prejudice and Discrimination Relating to Covid-19 in Bangladesh's Perspective. *Int. J. Integr. Educ.* 3, 138-145.doi: 10.31149/ijie.v3i9.605

Gopichandran, V., and Subramaniam, S. (2021). A qualitative inquiry into stigma among patients with Covid-19 in Chennai, India. *Indian J. Med. Ethics* 6, 193-201. doi: 10.20529/IJME.2021.013

Greene, T., Harju-Seppänen, J., Adeniji, M., Steel, C., Grey, N., Brewin, C. R., et al. (2021). Predictors and rates of PTSD, depression and anxiety in UK frontline health and social care workers during COVID-19. *Eur. J. Psychotraumatology* 12, 1882781. doi: 10.1080/20008198.2021.1882781

Grover, S., Sahoo, S., Mishra, E., Gill, K. S., Mehra, A., Nehra, R., et al. (2021). Fatigue, perceived stigma, self-reported cognitive deficits and psychological morbidity in patients recovered from COVID-19 infection. *Asian J. Psychiatr.* 64, 102815. doi: 10.1016/j.ajp.2021.102815

Haddad, C., Bou Malhab, S., Malaeb, D., Sacre, H., Saadeh, D., Mourtada, V., et al. (2021). Stigma toward people with COVID-19 among the Lebanese population: A cross-sectional study of correlates and mediating effects. *BMC Psychol.* 9, 164. doi: 10.1186/s40359-021-00646-y

Haft, S. L., and Zhou, Q. (2021). An outbreak of xenophobia: Perceived discrimination and anxiety in Chinese American college students before and during the COVID-19 pandemic. *Int. J. Psychol.* 56, 522–531. doi: 10.1002/ijop.12740

Hahm, H. C., Ha, Y., Scott, J. C., Wongchai, V., Chen, J. A., and Liu, C. H. (2021). Perceived COVID-19-related anti-Asian discrimination predicts post traumatic stress disorder symptoms among Asian and Asian American young adults. *Psychiatry Res.* 303, 114084. doi: 10.1016/j.psychres.2021.114084

Hahm, H. C., Xavier Hall, C. D., Garcia, K. T., Cavallino, A., Ha, Y., Cozier, Y. C., et al. (2021). Experiences of COVID-19-related anti-Asian discrimination and affective reactions in a multiple race sample of U.S. young adults. *BMC Public Health* 21, 1563. doi: 10.1186/s12889-021-11559-1

Harjana, N., Januraga, P. P., Indrayathi, P. A., Gesesew, H. A., and Ward, P. R. (2021). Prevalence of depression, anxiety, and stress among repatriated Indonesian migrant workers during the COVID-19 pandemic. *Front. Public Health* 9, 630295. doi: 10.3389/fpubh.2021.630295

He, J., He, L., Zhou, W., Nie, X., and He, M. (2020). Discrimination and social exclusion in the outbreak of COVID-19. *Int. J. Environ. Res. Public Health* 17, 2933. doi: 10.3390/ijerph17082933

He, L., Zhou, W., He, M., Nie, X., and He, J. (2021). Openness and COVID-19 induced xenophobia: The roles of trade and migration in sustainable development. *PloS ONE* 16, e0249579. doi: 10.1371/journal.pone.0249579

Hossain, M. B., Alam, M., Islam, M., Sultan, S., Faysal, M., Rima, S., et al. (2021). COVID-19 public stigma in the context of government-based structural stigma: A cross-sectional online survey of adults in Bangladesh. *Stigma Health* 6, 123-133. doi: 10.1037/sah0000305

Jassim, G., Jameel, M., Brennan, E., Yusuf, M., Hasan, N., and Alwatani, Y. (2021). Psychological Impact of COVID-19, isolation, and quarantine: A cross-sectional study. *Neuropsychiatr Dis. Treat.* 17, 1413–1421. doi: 10.2147/NDT.S311018

Jayakody, S., Hewage, S. A., Wickramasinghe, N. D., Piyumanthi, R. A. P., Wijewickrama, A., Gunewardena, N. S., et al. (2021). 'Why are you not dead yet?' - dimensions and the main driving forces of stigma and discrimination among COVID-19 patients in Sri Lanka. *J. Public Health* 199, 10–16. doi: 10.1016/j.puhe.2021.07.001

Jiang, T., Zhou, X., Lin, L., Pan, Y., Zhong, Y., Wang, X., et al. (2021). COVID-19-related stigma and its influencing factors: A nationwide cross-sectional study during the early stage of the pandemic in China. *BMJ Open* 11, e048983. doi: 10.1136/bmjopen-2021-048983

Kang, E., Lee, S. Y., Kim, M. S., Jung, H., Kim, K. H., Kim, K. N., et al. (2021). The psychological burden of COVID-19 stigma: Evaluation of the mental health of isolated mild condition COVID-19 patients. *J. Korean Med. Sci.* 36, e33. doi: 10.3346/jkms.2021.36.e33

Khan, S., Akter, S., Khan, T., Shariar, G., and Awal Miah, M. A. (2022). Psychological distress among Bangladeshi physicians: roles of perceived stigma, fear of infection and resilience in the context of Covid-19 pandemic. *J. Soc. Distress Homelessness* 31, 105-114. doi: 10.1080/10530789.2021.1892932

Khanal, P., Devkota, N., Dahal, M., Paudel, K., and Joshi, D. (2020). Mental health impacts among health workers during COVID-19 in a low resource setting: A cross-sectional survey from Nepal. *Glob. Health* 16, 89. doi: 10.1186/s12992-020-00621-z

Kirk, A. H., Chong, S. L., Kam, K. Q., Huang, W., Ang, L. S., Lee, J. H., et al. (2021). Psychosocial impact of the COVID-19 pandemic on paediatric healthcare workers. *Ann. Acad. Med. Singapore* 50, 203–211. doi: 47102/annals-acadmedsg.2020527

Labrague, L. J., De Los Santos, J., and Fronda, D. C. (2021). Perceived COVID-19-associated discrimination, mental health and professional-turnover intention among frontline clinical nurses: The mediating role of resilience. *Int. J. Ment. Health Nurs.* 30, 1674-1683. doi: 10.1111/inm.12920

Lee, S., and Waters, S. F. (2021). Asians and Asian Americans' experiences of racial discrimination during the COVID-19 pandemic: Impacts on health outcomes and the buffering role of social support. *Stigma Health* 6, 70-78. doi: 10.1037/sah0000275

Li, L., Wang, J., Leng, A., Nicholas, S., Maitland, E., and Liu, R. (2021). Will COVID-19 vaccinations end discrimination against COVID-19 patients in China? New evidence on recovered COVID-19 patients. *Vaccines* 9, 490. doi: 10.3390/vaccines9050490

Lin, B., Zhong, G., Liang, Z., Huang, J., Wang, X., and Lin, Y. (2021). Perceived-stigma level of COVID-19 patients in China in the early stage of the epidemic: A cross-sectional research. *PLoS ONE* 16, e0258042. doi: 10.1371/journal.pone.0258042

Litam, S. D. A., and Oh, S. (2020). Ethnic identity and coping strategies as moderators of COVID-19 racial discrimination experiences among Chinese Americans. *Couns. Outcome Res. Eval.* 13, 101-115. doi: 10.1080/21501378.2020.1814138

Litam, S. D. A., and Oh, S. (2021). Effects of COVID-19-related racial discrimination on depression and life satisfaction among young, middle, and older Chinese Americans. *Adultspan J.* 20, 70-84. doi: 10.1002/adsp.12111

Liu, D., Baumeister, R. F., Veilleux, J. C., Chen, C., Liu, W., Yue, Y., et al. (2020). Risk factors associated with mental illness in hospital discharged patients infected with COVID-19 in Wuhan, China. *Psychiatry Res.* 292, 113297. doi: 10.1016/j.psychres.2020.113297

Liu, Y., Finch, B. K., Brenneke, S. G., Thomas, K., and Le, P. D. (2020). Perceived discrimination and mental distress amid the COVID-19 pandemic: Evidence from the understanding America study. *Am. J. Prev. Med.* 59, 481-492. doi: 10.1016/j.amepre.2020.06.007

Lohiniva, A. L., Dub, T., Hagberg, L., and Nohynek, H. (2021). Learning about COVID-19-related stigma, quarantine and isolation experiences in Finland. *PLoS ONE* 16, e0247962. doi: 10.1371/journal.pone.0247962

Ma, H., and Miller, C. (2021). Trapped in a double bind: Chinese overseas student anxiety during the COVID-19 pandemic. *Health Commun.* 36, 1598-1605. doi: 10.1080/10410236.2020.1775439

Maglalang, D. D., Condor, J. L., Bañada, R., Nuestro, E., and Katigbak, C. (2021). Perceived discrimination and psychological distress: A survey of Filipinx Americans in Massachusetts during the COVID-19 pandemic. *Res. Sq.* [Preprint]. Available at: https://www.ncbi.nlm.nih.gov/pmc/articles/PMC8132238/ (Accessed June 1, 2022).

Mahmoudi, H., Saffari, M., Movahedi, M., Sanaeinasab, H., Rashidi-Jahan, H., Pourgholami, M., et al. (2021). A mediating role for mental health in associations between COVID-19-related self-stigma, PTSD, quality of life, and insomnia among patients recovered from COVID-19. *Brain Behav.* 11, e02138. doi: 10.1002/brb3.2138

Miconi, D., Li, Z. Y., Frounfelker, R. L., Santavicca, T., Cénat, J. M., Venkatesh, V., et al. (2021). Ethno-cultural disparities in mental health during the COVID-19 pandemic: a cross-sectional study on the impact of exposure to the virus and COVID-19-related discrimination and stigma on mental health across ethno-cultural groups in Quebec (Canada). *Bjpsych Open* 7, e14. doi: 10.1192/bjo.2020.146

Miconi, D., Li, Z. Y., Frounfelker, R. L., Venkatesh, V., and Rousseau, C. (2021). Socio-cultural correlates of self-reported experiences of discrimination related to COVID-19 in a culturally diverse sample of Canadian adults. *Int. J. Intercult. Relat.* 81, 176-192. doi: 10.1016/j.ijintrel.2021.01.013

Nursalam, N., Sukartini, T., Priyantini, D., Mafula, D., and Efendi, F. (2020). Risk factors for psychological impact and social stigma among people facing covid-19: A systematic review. *Syst. Rev. Pharm.* 11, 1022-1028. doi: 10.31838/srp.2020.6.146

Paleari, F. G., Pivetti, M., Galati, D., and Fincham, F. D. (2021). Hedonic and eudaimonic well-being during the COVID-19 lockdown in Italy: The role of stigma and appraisals. *Br. J. Health Psychol.* 26, 657–678. doi: 10.1111/bjhp.12508

Patel, B. R., Khanpara, B. G., Mehta, P. I., Patel, K. D., and Marvania, N. P. (2021). Evaluation of perceived social stigma and burnout, among health-care workers working in covid-19 designated hospital of India: A cross-sectional study. *Asian J. Soc. Health Behav.* 4, 156-162. doi: 10.4103/shb.shb_54_21

Perry, S. L., Whitehead, A. L., and Grubbs, J. B. (2021). Prejudice and pandemic in the promised land: How white Christian nationalism shapes Americans' racist and xenophobic views of COVID-19. *Ethnic Racial Studies* 44, 759-772. doi: 10.1080/01419870.2020.1839114

Poyraz, B. Ç., Poyraz, C. A., Olgun, Y., Gürel, Ö., Alkan, S., Özdemir, Y. E., et al. (2021). Psychiatric morbidity and protracted symptoms after COVID-19. *Psychiatry Res.* 295, 113604. doi: 10.1016/j.psychres.2020.113604

Radhakrishnan, R. V., Jain, M., Mohanty, C. R., Jacob, J., Shetty, A. P., Stephen, S., et al. (2021). The perceived social stigma, self-esteem, and its determinants among the health care professionals working in India during COVID 19 pandemic. *Med. J. Armed Forces India* 77, S450-S458. doi: 10.1016/j.mjafi.2021.01.017

Ransing, R., Ramalho, R., de Filippis, R., Ojeahere, M. I., Karaliuniene, R., Orsolini, L., et al. (2020). Infectious disease outbreak related stigma and discrimination during the COVID-19 pandemic: Drivers, facilitators, manifestations, and outcomes across the world. *Brain, Behav., Immun.* 89, 555-558. doi: 10.1016/j.bbi.2020.07.033

Rzymski, P., and Nowicki, M. (2020). COVID-19-related prejudice toward Asian medical students: A consequence of SARS-CoV-2 fears in Poland. *J. Infect. Public Health* 13, 873-876. doi: 10.1016/j.jiph.2020.04.013

Saeed, F., Mihan, R., Mousavi, S. Z., Reniers, R. L., Bateni, F. S., Alikhani, R., et al. (2020). A narrative review of stigma related to infectious disease outbreaks: What can be learned in the face of the Covid-19 pandemic? *Front. Psychiatry* 11, 565919. doi: 10.3389/fpsyt.2020.565919

Sahoo, B. P., and Patel, A. B. (2021). Social stigma in time of COVID-19 pandemic: Evidence from India. *Int. J. Sociol. Soc. Policy* 4, 1170-1182. doi: 10.1108/IJSSP-01-2021-0012

Schmidt, T., Cloete, A., Davids, A., Makola, L., Zondi, N., and Jantjies, M. (2020). Myths, misconceptions, othering and stigmatizing responses to Covid-19 in South Africa: A rapid qualitative assessment. *PloS ONE* 15, e0244420. doi: 10.1371/journal.pone.0244420

Shiu, C., Chen, W. T., Hung, C. C., Huang, E. P., and Lee, T. S. (2022). COVID-19 stigma associates with burnout among healthcare providers: Evidence from Taiwanese physicians and nurses. *J. Formos. Med. Assoc.* 121, 1384-1391. doi: 10.1016/j.jfma.2021.09.022

Singh, R., Subedi, M., Sunar, C. B., Pant, S., Singh, B., Shah, B., et al. (2021). Association of social stigma of COVID-19 with work satisfaction, burnout and fatigue among healthcare workers in Nepal. *Glob. Psychiatry Arch.* 4, 180-190 doi: 10.52095/gp.2021.3838.1027

Sorokin, M. Y., Kasyanov, E. D., Rukavishnikov, G. V., Makarevich, O. V., Neznanov, N. G., Morozov, P. V., et al. (2020). Stress and stigmatization in health-care workers during the COVID-19 pandemic. *Indian J. Psychiatry* 62, S445-S453. doi: 10.4103/psychiatry.IndianJPsychiatry_870_20

Taylor, S., Landry, C. A., Rachor, G. S., Paluszek, M. M., and Asmundson, G. (2020). Fear and avoidance of healthcare workers: An important, under-recognized form of stigmatization during the COVID-19 pandemic. *J. Anxiety Disord.* 75, 102289 doi: 10.1016/j.janxdis.2020.102289

Teksin, G., Uluyol, O. B., Onur, O. S., Teksin, M. G., and Ozdemir, H. M. (2020). Stigma-related factors and their effects on health-care workers during COVID-19 pandemics in Turkey: A multicenter study. *Med. Bull. Sisli Etfal Hosp.* 54, 281–290. doi: 10.14744/SEMB.2020.02800

Tsai, J. Y., Phua, J., Pan, S., and Yang, C. C. (2020). Intergroup contact, COVID-19 news consumption, and the moderating role of digital media trust on prejudice toward Asians in the United States: Cross-sectional study. *J. Med. Internet Res.* 22, e22767. doi: 10.2196/22767

Uvais, N. A., Shihabudheen, P., Bishurul, N. A., and Moideen, S. (2021). COVID-19-related stigma and stress among doctors working in the private sector during the COVID-19 pandemic. *Prim. Care Companion CNS Disord.* 23, 36614. doi: 10.4088/PCC.21br02956

Wang, S., Chen, X., Li, Y., Luu, C., Yan, R., and Madrisotti, F. (2020). 'I'm more afraid of racism than of the virus!': racism awareness and resistance among Chinese migrants and their descendants in France during the Covid-19 pandemic. *Euro. Soc.* 23, S721-S742. doi: 10.1080/14616696.2020.1836384

Wu, C., Qian, Y., and Wilkes, R. (2021). Anti-Asian discrimination and the Asian-white mental health gap during COVID-19. *Ethnic Racial Studies* 44, 819-835. doi: 10.1080/01419870.2020.1851739

Xin, M., Luo, S., She, R., Yu, Y., Li, L., Wang, S., et al. (2020). Negative cognitive and psychological correlates of mandatory quarantine during the initial COVID-19 outbreak in China. *Am. Psychol.* 75, 607–617. doi: 10.1037/amp0000692

Xu, J., Sun, G., Cao, W., Fan, W., Pan, Z., Yao, Z., et al. (2021). Stigma, discrimination, and hate crimes in Chinese-speaking world amid Covid-19 pandemic. *Asian J. Criminol.* 16, 51–74. doi: 10.1007/s11417-020-09339-8

Yadav, K., Laskar, A. R., and Rasania, S. K. (2020). A study on stigma and apprehensions related to COVID-19 among healthcare professionals in Delhi. *Int. J. Community Med. Public Health* 7, 4547-4553. doi: 10.18203/2394-6040.ijcmph20204760

Yang, C. C., Tsai, J. Y., and Pan, S. (2020). Discrimination and well-being among Asians/Asian Americans during COVID-19: The role of social media. *Cyberpsychol. Behav. Soc. Netw.* 23, 865-870. doi: 10.1089/cyber.2020.0394

Yang, F. X., and Wong, I. A. (2020). The social crisis aftermath: Tourist well-being during the COVID-19 outbreak. *J. Sustain. Tour.* 29, 859-878. doi: 10.1080/09669582.2020.1843047

Yu, N., Pan, S., Yang, C. C., and Tsai, J. Y. (2020). Exploring the role of media sources on COVID-19-related discrimination experiences and concerns among Asian people in the United States: Cross-sectional survey study. *J. Med. Internet Res.* 22, e21684. doi: 10.2196/21684

Yuan, Y., Zhao, Y. J., Zhang, Q. E., Zhang, L., Cheung, T., Jackson, T., et al. (2021). COVID-19-related stigma and its sociodemographic correlates: a comparative study. *Glob. Health* 17, 54. doi: 10.1186/s12992-021-00705-4

Yufika, A., Pratama, R., Anwar, S., Winardi, W., Librianty, N., Prashanti, N. A. P., et al. (2021). Stigma associated with COVID-19 among health care workers in Indonesia. *Disaster Med. Public Health Preparedness* doi: 10.1017/dmp.2021.93

Zandifar, A., Badrfam, R., Khonsari, N. M., Mohammadi, M. R., Asayesh, H., and Qorbani, M. (2020). Prevalence and associated factors of posttraumatic stress symptoms and stigma among health care workers in contact with COVID-19 patients. *Iran. J. Psychiatry* 15, 340–350. doi: 10.18502/ijps.v15i4.4303

Zhang, T. M., Fang, Q., Yao, H., and Ran, M. S. (2021). Public stigma of COVID-19 and its correlates in the general population of China. *Int. J. Environ. Res. Public Health* 18, 11718. doi: 10.3390/ijerph182111718

Zolnikov, T. R., and Furio, F. (2020). Stigma on first responders during COVID-19. *Stigma Health* 5, 375-379. doi: 10.1037/sah000027
